# An Efficient Site-Specific Method for Irreversible Covalent Labeling of Proteins with a Fluorophore

**DOI:** 10.1038/srep16883

**Published:** 2015-11-19

**Authors:** Jiaquan Liu, Jeungphill Hanne, Brooke M. Britton, Matthew Shoffner, Aaron E. Albers, Jared Bennett, Rachel Zatezalo, Robyn Barfield, David Rabuka, Jong-Bong Lee, Richard Fishel

**Affiliations:** 1 Department of Molecular Virology, Immunology and Medical Genetics, The Ohio State University Medical Center, Columbus, OH 43210; 2 Catalent Biologics–West, Emeryville, CA 94608; 3 Department of Physics, Pohang University of Science and Technology (POSTECH), Pohang, Korea; 4 School of Interdisciplinary Bioscience and Bioengineering, POSTECH, Pohang, Korea; 5 Physics Department, The Ohio State University, Columbus, OH 43210

## Abstract

Fluorophore labeling of proteins while preserving native functions is essential for bulk Förster resonance energy transfer (FRET) interaction and single molecule imaging analysis. Here we describe a versatile, efficient, specific, irreversible, gentle and low-cost method for labeling proteins with fluorophores that appears substantially more robust than a similar but chemically distinct procedure. The method employs the controlled enzymatic conversion of a central Cys to a reactive formylglycine (fGly) aldehyde within a six amino acid Formylglycine Generating Enzyme (FGE) recognition sequence *in vitro*. The fluorophore is then irreversibly linked to the fGly residue using a Hydrazinyl-Iso-Pictet-Spengler (HIPS) ligation reaction. We demonstrate the robust large-scale fluorophore labeling and purification of *E.coli* (Ec) mismatch repair (MMR) components. Fluorophore labeling did not alter the native functions of these MMR proteins *in vitro* or *in singulo*. Because the FGE recognition sequence is easily portable, FGE-HIPS fluorophore-labeling may be easily extended to other proteins.

FRET and single molecule fluorescence tracking have become versatile tools in modern molecular biology^1^,^2^. Use of these techniques has greatly improved our understanding of many biophysical processes including replication^3^,^4^,^5^,^6^,^7^, transcription^8^,^9^,^10^,^11^,^12^,^13^,^14^, translation^15^,^16^,^17^ and DNA repair^18^,^19^,^20^,^21^,^22^. These studies generally employ fluorescent molecules as an imaging tool^3^,^4^,^5^,^8^,^9^,^10^,^11^,^15^,^16^,^19^,^20^,^21^. A common fluorescence imaging technique employs quantum dot (QD) labeling. However, the size of the QDs (10–50 nm) can often exceed the size of the molecule that is being imaged. These issues may lead to unusual solution and diffusion characteristics of QD-labeled proteins. Moreover, detection of molecular interactions using FRET between appropriate QD excitation-emission pairs is inherently inefficient^23^. In contrast, numerous small chemical fluorophores display both high quantum yield and FRET efficiency.

Conventional methods employed for flourophore-labeling of proteins often impact native function(s). This is especially true in the case of more chemically sensitive protein targets. A number of protein-fluorophore labeling methods have been reported including: Cys-maleimide chemistry, incorporation of non-natural reactive amino acids as well as peptide tags such as Halo(haloalkane dehalogenase), SNAP/CLIP(O^6^-alkylguanine-DNA alkyltransferase), Avi(biotin ligase recognition peptide), Sfp phosphopantetheinyl transferase(CoA), Sortase and others^24^. However, there are important limitations associated with these methods. For example, Cys-maleimide conjugation requires a single Cys residue located in a benign structural position of the protein target. Other methods suffer from low labeling efficiencies, require expensive reagents or result in abnormally large fluorophore-protein complexes^24^.

Recently, a site-specific conjugation method was described that relies on the incorporation of a six amino acid FGE recognition sequence, Leu-Cys-Thr-Pro-Ser-Arg (LCTPSR). Conversion of the central Cys residue to an fGly produces a reactive aldehyde that may then be used for chemical coupling^25^,^26^,^27^. Co-expression of FGE with LCTPSR-containing target proteins appeared to catalyze Cys → fGly conversion *in vivo* permitting chemical coupling of a hydrazide-modified fluorophore^1^. Although substantial fluorophore labeling was reported a number of technical issues arose that included: (1) the use of large quantities of expensive hydrazide-modified dyes (75.6 mM; 60 mg Cy3/ml) to obtain extensive labeling, (2) the conversion of Cys to fGly *in vivo* was not quantified, (3) the specificity of fluorophore labeling to the fGly residue was not determined, and (4) the effects of the labeling process on overall protein specific-activity was not fully determined.

Here we describe a vastly improved FGE-based fluorophore labeling method. The protocol relies on efficient and controlled FGE conversion of Cys to fGly *in vitro* followed by specific and irreversible fluorophore labeling using the Hydrazinyl-Iso-Pictet-Spengler (HIPS) ligation method. Labeling requires ~150-fold less fluorophore and may be performed under mild solution conditions. We demonstrate efficient, site-selective, and large-scale preparation fluorophore-labeling of relatively labile EcMMR components that retained high specific activity. The portability of the FGE recognition sequence should make HIPS-fluorophore labeling widely applicable for single molecule imaging experiments as well as bulk and kinetic FRET interaction studies.

## Results

MMR is an excision-resynthesis reaction that repairs mismatched nucleotides that arise primarily as a result of polymerase misincorporation errors^28^. The initial recognition of mismatched nucleotide is carried out by MutS homologs (MSHs)^29^,^30^,^31^. MSH proteins form a long-lived mismatch-provoked ATP-bound sliding clamp that recruits MutL homologs (MLH/PMS)^32^; ultimately authorizing strand-specific excision and repair. The majority of single molecule MMR studies have used a singlet-Cys *Thermus aquaticus* TaMutS labeled with a maleimide-functionalized fluorophore^19^,^21^. Single molecule imaging of EcMutS and EcMutL (as well as other MSH and MLH/PMS proteins) is correspondingly difficult since they contain multiple structurally essential Cys residues.

Based on the prototypical FGE-based fluorophore labeling method described by Shi *et al.*^1^, we modified the largely disordered C-terminus of EcMutS to contain tandem hexa-histidine (his_6_) and FGE (LCTPSR; ald_6_) tags (EcMutS-his_6_/ald_6_; Table S1). The his_6_ was separated from the EcMutS C-terminus by two Ser residues and the ald_6_ was separated from the his_6_ by two Gly residues. The EcMutS-his_6_/ald_6_ was shown to genetically suppress the elevated mutation rates associated with *E.coli* Δ*mutS* mutator phenotype ensuring that the tags did not interfere with *wild type* activities (Fig. S1). Two compatible plasmids were constructed to simultaneously express EcMutS (pET29a backbone) and *Mycobacterium tuberculosis* MtFGE (pBAD42 backbone)^1^. The EcMutS-his_6_/ald_6_ was enriched using a Ni-NTA column, labeled with Cy3-hydrazide fluorophore^1^ and free-dye removed using Heparin column chromatography. MonoQ chromatography resulted in >95% purified EcMutS-his_6_/ald_6_. We observed ~1% fluorophore-labeled protein in the presence of 4 mM Cy3-hydrazide (Fig. S2A), which increased to 5% fluorophore-labeled protein with 13 mM Cy3-hydrazide (Fig. S2B). When we increased the Cy3-hydrazide dye concentration to 66 mM, which was below the 75.6 mM dye concentration recommended by Shi *et al.*^1^, we observed ~30% fluorophore-labeled protein. However, virtually all of the EcMutS was insoluble under these conditions and became refractory to further purification (Fig. S3A). A similar precipitation propensity was observed when EcMutL-his_6_/ald_6_, EcRecJ-his_6_/Ald_6_ and HsMSH2-ald_6_-HsMSH6-his_6_ containing virtually identical his_6_/ald_6_ tags labeled with Cy3- or Cy5-hydrazide (Fig. S3A; data not shown). We altered the central Cys residue to Ala in the ald_6_-tag [EcMutS-his_6_/ald_6_(C865A); Table S1] to examine the specificity of Cy3-hydrazide (66 mM) fluorophore labeling. We found that only 30% of the fluorophore-labeled EcMutS-his_6_/ald_6_ protein could be considered specifically linked to the fGly residue (~9% of the total protein; Fig. S3B). These results suggested that the high concentrations of hydrazide-dyes induced solution instability of EcMutS and that hydrazide-fluorophore labeling of the ald_6_-tagged MMR protein was largely non-specific.

Previous observations have suggested that the labeling efficiency of hydrazide-functionalized fluorophores might be compromised by the low equilibrium constants associated with hydrazone formation in solution^33^. Moreover, the instability of the hydrazone bond results in shortened half-lives for hydrazone-labeled proteins^34^. In contrast, the Hydrazinly-Iso-Pictet-Spengler (HIPS) ligation reaction has been shown to produce stable and irreversible covalent conjugates with reactive aldehydes at neutral pH^35^,^36^.

**Figure 1:**
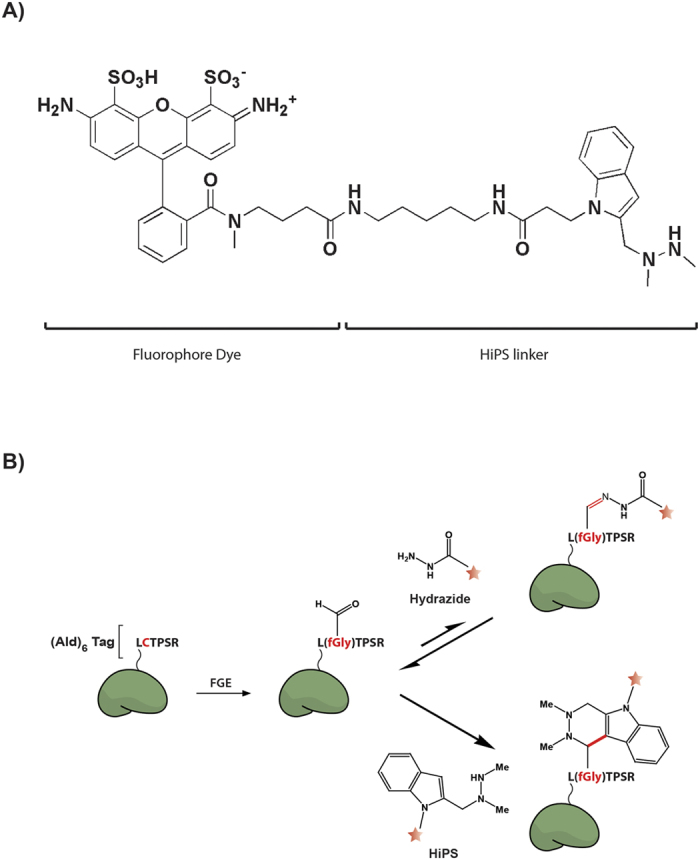
HIPS-fluorophore chemical structure. **(A)** Structure of the Atto488-Hydrazino-Pictet-Spengler (HIPS) fluorophore showing the Dye and HIPS linker. **(B)** The reaction scheme for the ald_6_-protein with the Hydrazide or HiPS-dyes.

**Figure 2:**
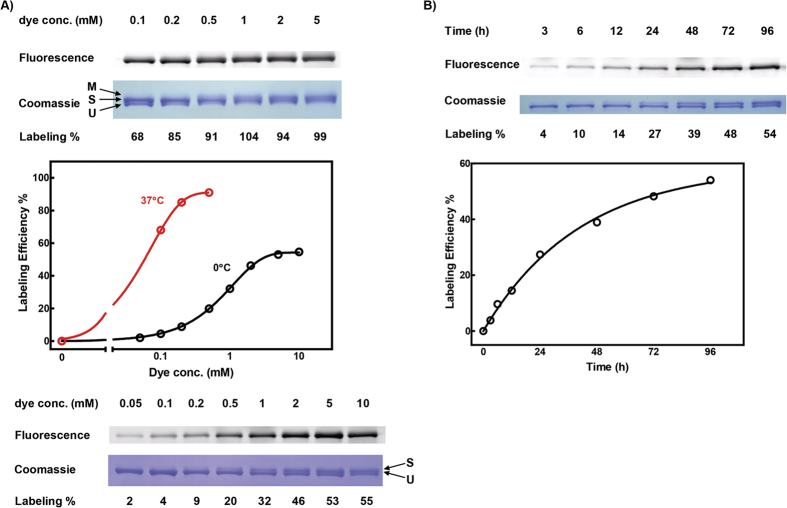
HIPS-fluorophore labeling analysis. Maltose Binding Protein (MBP) containing an FGE recognition sequence in which ~99% of the central cystein was converted to fGly (MBP-ald_6_) was used to determine labeling efficiency^26^. **(A)** (top panels) fluorophore dye concentration, the fluorescence scan of the PAGE gel and the coomassie stained PAGE gel of MBP-ald_6_ following HIPS ligation at 37 °C (graphed in middle panel). The coomassie stained PAGE gel shows the location of the unlabeled (U), single-labeled (S) and multiply labeled (M) HIPS-fluorophore. We noted that above 0.2 mM HIPS-dye at 37 °C the quantity of protein that was labeled with more than one dye became significant reducing the quantification accuracy of specific labeling to the FGE-converted fGly. (bottom panels) the fluorophore dye concentration, the fluorescence scan of the PAGE gel and the coomassie stained PAGE gel of MBP-ald_6_ following HIPS ligation at 0 °C (graphed in middle panel). **(B)** Kinetics of HIPS-dye labeling to MBP-ald_6_. Top panels show time of incubation, the fluorescence scan of the PAGE gel and the coomassie stained PAGE gel of MBP-ald_6_ following HIPS ligation at 0 °C. Labeling efficiency was calculated as described in the Materials and Methods and accounts for loading variations between lanes. The Fluorescent scans and Coomassie stained gels have been cropped to show only the relevant protein bands, which in these studies accounts for >80% of the visible bands.

We conjugated a HIPS linker to cadaverine-modifed Alexa-Fluor (AF) fluorophores (AF488, AF555, AF594 and AF647) as well as NHS-ester modified Atto488 similar to a previously described procedure (Fig. 1; Supplementary Materials and Methods)^36^. Partially purified Maltose Binding Protein containing an ald_6_-tag (MBP-ald_6_) was used as a fluorophore-labeling target. Mass spectroscopic (MS) analysis suggested that the ratio of fGly:Cys in the MBP-ald_6_ preparation was 99:1, and that ~80% of these fGly residues could be linked to a HIPS-fluorophore^26^. However, MS may not detect FGE-converted Cys residues that have been subsequently altered or degraded to non-reactive chemical forms. We found that fluorophore conjugation to the MBP-ald_6_ substrate induced a visible molecular weight shift in SDS-PAGE gels that allowed easy quantification of unlabeled (U) and specifically labeled (S) protein (Fig. 2A). Using this assay we determined that the absolute reactivity of the MBP-ald_6_ substrate under identical solution conditions to our previous studies^26^ was initially linear and saturated at 85% total labeling at 37 °C (Fig. 2A, red). We noted higher molecular weight bands (M) at fluorophore concentrations above 0.2 mM, suggesting non-specific fluorophore labeling as the specific fGly-fluorophore linking approached saturation.

Because of the comparative instability of MMR proteins we wished to examine fluorophore labeling at 0 °C where these proteins may retain maximum activity over several days. At 0 °C we found that MBP-ald_6_ labeling saturated at 55% total labeling (Fig. 2A, black). The lower level of labeling saturation at 0 °C compared to 37 °C likely reflects different equilibrium dynamics. Saturation of fluorophore labeling at 0 °C occurred between 2–5 mM HIPS-AF647 after 48 h (Fig. 2A, black) and at 96 h with 2 mM HIPS-AF647 (Fig. 2B). These HIPS-based fluorophore-labeling observations provided well-defined experimental windows to explore efficient labeling of MMR proteins.

**Figure 3:**
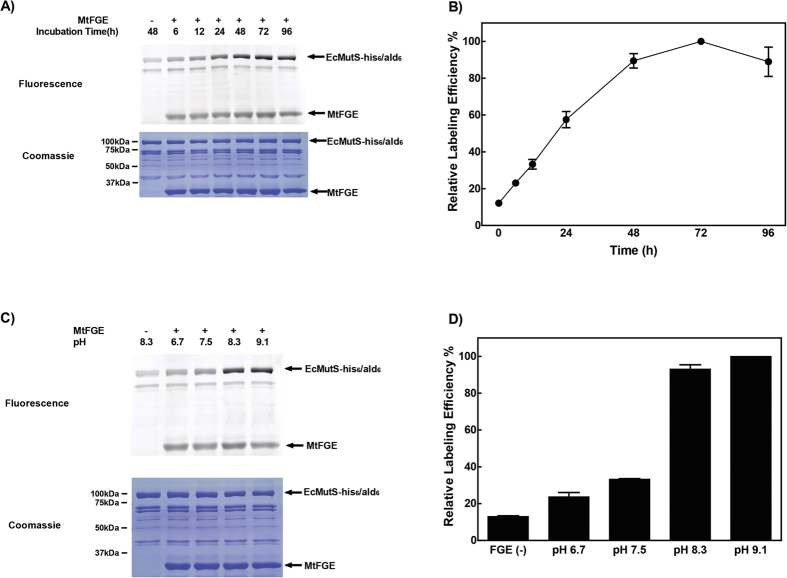
FGE conversion *in vitro* enhances HIPS-fluorophore labeling. **(A)** Fluorescent scan and coomassie stained gel of the FGE conversion *in vitro* kinetics using 2 mM HIPS-Atto488. **(B)** The fluorescent signal relative to the coomassie signal was quantified (Molecular Dynamics Image Quant), followed by setting the maximum ratio in the analysis to 100% to normalized the relative labeling efficiency of EcMutS-his_6_/ald_6_ (see Material and Methods). **(C)** Fluorescent scan and coomassie stained gel of the pH-dependence of FGE conversion *in vitro* using 2 mM HIPS-Atto488. **(D)** The fluorescent signal relative to the coomassie signal was quantified (Molecular Dynamics Image Quant), followed by setting the maximum ratio in the analysis to 100% to normalized the relative labeling efficiency of EcMutS-his_6_/ald_6_ (see Material and Methods).

**Figure 4:**
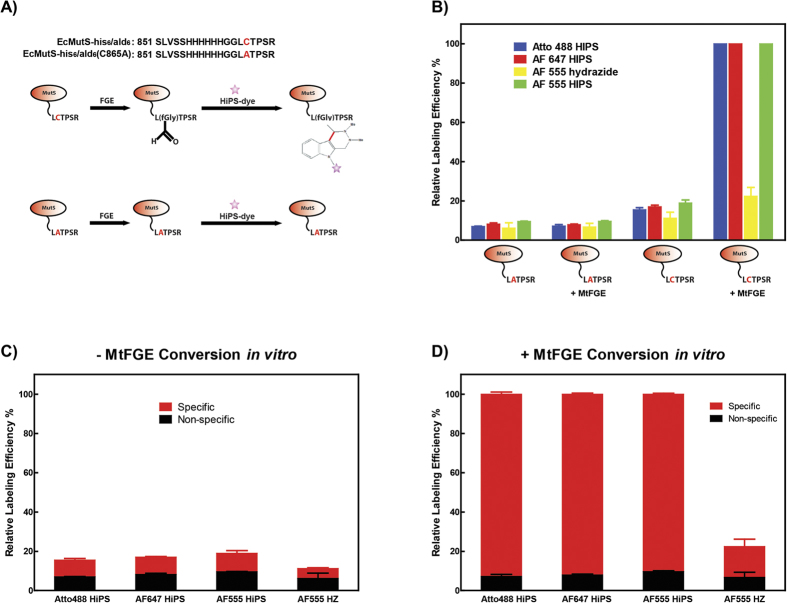
HIPS-fluorophore ligation to fGly within an FGE site is highly specific. **(A)** C-terminal sequences of EcMutS-his_6_/ald_6_ and EcMutS-his_6_/ald_6_(C865A) as well as an illustration of HIPS-FGE labeling. **(B)** The normalized relative labeling efficiency (%) was calculated from Fig. S7 for EcMutS-his_6_/ald_6_(C865A) in the absence of MtFGE conversion *in vitro*, EcMutS-his_6_/ald_6_(C865A) following MtFGE conversion *in vitro* for 48 hrs, EcMutS-his_6_/ald_6_ in the absence of MtFGE conversion *in vitro*, and EcMutS-his_6_/ald_6_ following MtFGE conversion *in vitro* for 48 hrs each in the using the Atto488-HIPS (blue), AF647-HIPS (red), AF555-hydrazide (yellow), and AF555-HIPS fluorophore dyes (green). **(C)** The specificity of EcMutS-his_6_/ald_6_ fluorophore-labeling in the absence of MtFGE conversion *in vitro*. Relative labeling efficiency (%) of EcMutS-his_6_/ald_6_(C865A) (black) is considered non-specific and the relative labeling efficiency (%) of EcMutS-his_6_/ald_6_ minus the relative labeling efficiency (%) of EcMutS-his_6_/ald_6_(C865A) is considered specific for the FGE site (red). **(D)** The specificity of EcMutS-his_6_/ald_6_ fluorophore-labeling in the presence of MtFGE conversion *in vitro* for 48 hrs. Specific and non-specific labeling were calculated as in panel (**C**).

The fGly conversion of the ald_6_-tag *in vivo* may vary between different protein substrates^1^,^26^,^37^. We found lower expression levels of MtFGE when co-expressed with EcMutS (Fig. S4A) as well as insolubility when MtFGE was expressed alone (Fig. S4B). These expression issues appeared to greatly attenuate the conversion reaction *in vivo*. Moreover, there are multiple other cellular enzymes that may catalyze the chemical modification of aldehydes *in vivo*^38^,^39^,^40^ resulting in an obligate reduction in reactivity. As an alternative, we developed an FGE conversion step *in vitro* as an approach to control and retain fGly reactive aldehydes^27^,^41^. To examine the conversion efficiency with MMR proteins, EcMutS-his_6_/ald_6_ was partially purified using Ni-NTA and incubated with partially purified his_6_-FGE at a ratio of 1:1 (7 μM ea) for varying times at 4 °C (Fig. 3A,B). We observed a near linear relationship between the MtFGE incubation time and the relative labeling efficiency of EcMutS-his_6_/ald_6_ with HIPS-Atto488 (2 mM) up to 48 h that was followed by reaction saturation. The use of a 1:1 ratio of MtFGE to target MMR protein appears to suggest that the conversion reaction is not catalytic. However, we performed the fGly conversion reaction at 4^o^C where turnover of the enzyme is known to be quite slow^42^. When conversion is performed at higher temperatures, the reaction becomes catalytic with ratios of target to FGE of 100-1000:1^42^. Interestingly, the labeling kinetics of EcMutS-his_6_/ald_6_ with HIPS-AF555 (0.4 mM) was rapid and non-linear for the first 3 h to ~20% labeling followed by an apparently linear slower kinetics up to 72 h (Fig. S5A,B). However, subtraction of the 10% “nonspecific” labeling (see Fig. 4D) from each time-point results in a labeling curve (Fig. S5C), that appeared similar to the MBP-ald_6_ labeling curve (Fig. 2B). We also noted fluorophore labeling of the MtFGE, which has been ascribed to auto-conversion^43^.

We examined the pH dependence of fGly conversion (Fig. 3C,D). While fluorophore-labeling appeared slightly greater at pH 9.1, we determined that the optimum pH for sufficient EcMutS-his_6_/ald_6_ conversion that fully preserved the enzyme activity was pH 8.3. To examine the general applicability of our method we introduced an ald_6_-tag onto the C-terminus of EcMutL (EcMutL-his_6_/ald_6_) and an internal site of EcMutL [EcMutL(346 ald_6_)-his_6_] that genetically complemented isogenic Δ*mutL* (Fig. S1). In addition, we examined the labeling efficiency of the 5′ → 3′ MMR exonuclease EcRecJ (EcRecJ-his_6_/ald_6_). We found FGE-dependent conversion and labeling that was clearly specific compared to contaminating peptides in the partially purified MMR protein fractions (Fig. S6). Interestingly, we found that SDS-PAGE could separate labeled from unlabeled EcMutL monomer (Fig. S6C). Using simple Gaussian fits we determined that that 35% of the [EcMutL(346 ald_6_)-his_6_] appeared to be singly labeled with AF647.

The specificity of HIPS-fluorophore and Hydrazide-fluorophore labeling was quantitatively examined using the EcMutS-his_6_/ald_6_(C865A) substitution mutation (Fig. 4A; Fig. S7A). In the absence of FGE conversion *in vitro* we observed dramatically reduced fluorophore labeling of EcMutS-his_6_/ald_6_, which was further reduced at least 2-fold with the EcMutS-his_6_/ald_6_(C865A) substitution mutation (Fig. 4B; Fig. S7A). This labeling trend was consistent for three different HIPS-modified AF fluorophores (Fig. 4B; Fig. S7A). As a control we found that the labeling efficiency using hydrazide-modified AF555 was reduced an additional 2–3 fold compared to labeling with HIPS-modified AF fluorophores (Fig. 4B, yellow). Following FGE conversion *in vitro* the labeling efficiency of the HIPS-modified fluorophores increased 8–10 fold (Fig. 4B; Fig. S7A), while the labeling efficiency of the hydrazide-modified AF555 increased no more than 2-fold. We also demonstrate that the AF-HiPS dyes are stable and insensitive to SDS and boiling during sample preparation (compare Fig. S7A,B). These results are consistent with our previous conclusion that FGE conversion *in vitro* significantly enhances HIPS-modified fluorophore labeling efficiency. In addition, the hydrazide-modified fluorophores display substantially reduced labeling efficiency compared to HIPS-modified fluorophores.

In the absence of FGE conversion *in vitro*, <50% of the fluorophore label was specific for the fGly within the ald_6_-tag (Fig. 4C; Fig. S7). Moreover, the specificity of the hydrazide-modified AF555 in the absence of FGE conversion *in vitro* was near background. In the presence of FGE conversion *in vitro* the labeling efficiency was >90% specific for the fGly within the ald_6_-tag (Fig. 4D). In contrast, even with extreme excess of fluorophore the hydrazide-modified AF555 exhibited >5-fold less relative labeling in which at least 30% was not specific for the fGly within the ald_6_-tag (Fig. 4D; Fig. S7). Taken as a whole, these results suggest that combining FGE conversion of the ald_6_-tag *in vitro* followed by labeling with HIPS-modified fluorophores dramatically enhanced labeling efficiency and specificity.

We examined the stability of the HIPS-fluorophore conjugate to EcMutS-his_6_/ald_6_ (Fig. S8A,B). Following incubation at 25 °C for 24 h we detected less than 0.7% loss of fluorophore (Fig. S8A,B). While the stability of a hydrazone-fluorophore conjugate could not be directly examined due to low labeling efficiency and solution instability, the stability of a related hydroxylamine-aldehyde conjugation that forms an aminooxy-aldehyde was determined (Fig. S8C). We found that 31% of the aminooxy-aldehyde fluorophore linkage was lost after 24 h at 37 °C (Fig. S8C). Moreover, 63% of the aminooxy-aldehyde conjugated fluorophore was lost after 6 d, while the HIPS-aldehyde lost only 16% after 6 d at 37 °C (Fig. S8C). It is important to note that the aminooxy-aldehyde conjugation has been reported to be to be far more stable than the hydrazide/hydrazone-aldehyde bond^34^, suggesting that HIPS-conjugated fluorophores are significantly more stable than hydrazide-conjugated fluorophores.

**Figure 5:**
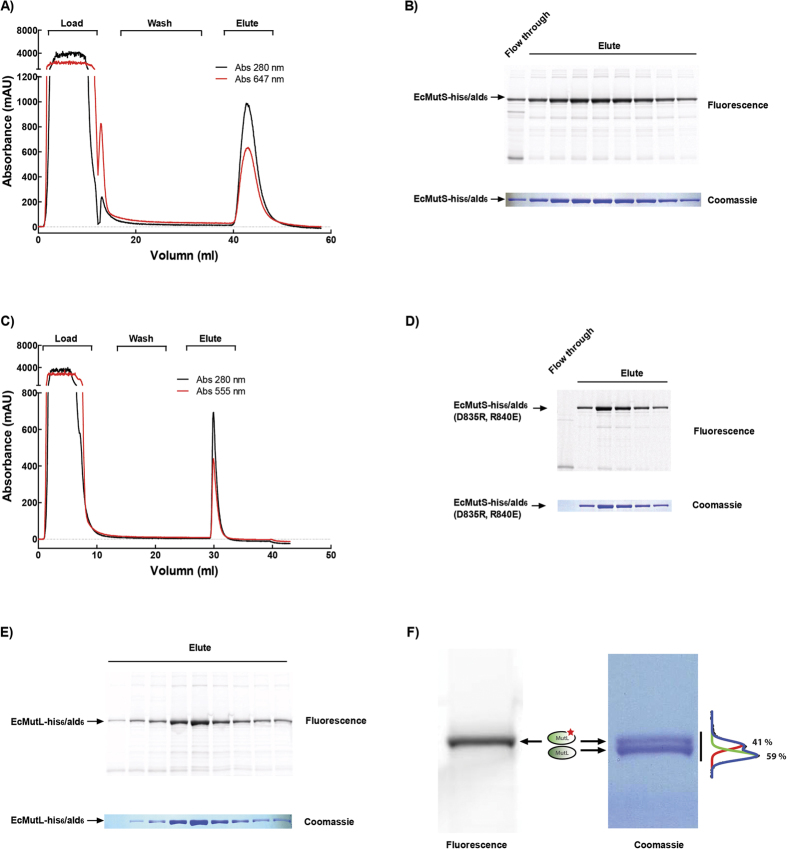
HIPS fluorophore labeling and purification of EcMutS-his_6_/ald_6_ and EcMutL-his_6_/ald_6_. **(A,B)** HIPS-AF647 fluorophore-labeling and purification of EcMutS-his_6_/ald_6_. Final heparin chromatography (**A**) and Fluorescence scan (top) and coomassie stain (bottom) of eluted fractions separated using SDS-PAGE gel (**B**). **(C,D)** HIPS-AF555 fluorophore-labeling and purification of EcMutS-his_6_/ald_6_(D835R,R840E). Final heparin chromatography (**C**) and Fluorescence scan (top) and coomassie stain (bottom) of eluted fractions separated using SDS-PAGE gel (**D**). **(E,F)** HIPS-AF647 fluorophore-labeling and purification of EcMutL-his_6_/ald_6_. Fluorescence scan (top) and coomassie stain (bottom) of eluted fractions separated using SDS-PAGE gel (**E**) and fluorescence scan (left) and coomassie stain (right) with Gaussian fitting of labeled (red) and unlabeled (green) EcMutL-his_6_/ald_6_ (**F**). The Coomassie stained gels have been cropped to show only the relevant protein bands, which in these studies accounts for >90% of the visible bands.

The lack of efficient and specific fluorophore labeling protocols has limited the rigorous examination of bulk and single molecule kinetic interactions between MMR proteins. Since EcMutS and EcMutL largely exist as stable dimers^44^,^45^, we calculated that 30% monomer labeling would result in 9% containing two fluorophores. Based on the MBP-ald_6_ data (Fig. 2A,B), we performed FGE-HIPS fluorophore labeling using 0.5 mM HIPS-AF647 (4.8 mg) with 40 μM (30 mg) of EcMutS-his_6_/ald_6_ and 0.5 mM HIPS-AF555 (0.6 mg) with 15 μM (1.5 mg) EcMutS-his_6_/ald_6_(D835R,R840E). The EcMutS(D835R,R840E) substitution mutations eliminate interaction between EcMutS dimers *in vitro* but do not appear to affect MMR-dependent mutation suppression *in vivo*^46^. The unreacted fluorophore and FGE were removed by Heparin chromatography resulting in a >95% purified protein (Fig. 5A–D). The final labeling efficiency was determined to be 23% with the EcMutS-his_6_/ald_6_ (16 μM) and 34% with the EcMutS-his_6_/ald_6_(D835R,R840E) (11 μM; Fig. 5A–D; Fig. S9A,B; Table S2). Since there does not appear to be an absolute correlation with the ratio of fluorophore to protein concentration for these two EcMutS constructs, we ascribe the modest differences in labeling efficiency to ald_6_-tag accessibility during conversion and/or labeling.

We labeled and purified EcMutL-his_6_/ald_6_ following the same protocol we developed for EcMutS-his_6_/ald_6_ with minor modifications (Fig. 5E–F; Materials and Methods). We obtained 35% AF647 fluorophore-labeled monomer EcMutL-his_6_/ald_6_ (18 μM), which translates to a calculated 46% of singly labeled dimers with an additional 12% of the dimers containing two-fluorophores (Fig. S9C; Table S2). We determined that 41% of the EcMutL appeared to be singly labeled with AF647 by SDS-PAGE analysis (Fig. 5F), which appears similar to the spectrophotometry measure (Fig. S9C). While there may be modest differences in labeling efficiency between MMR proteins, preparations and ald_6_-tag location within a peptide, these results suggest that the labeling curves generated with the MBP-ald_6_ can be generalized to most ald_6_-tagged proteins. Taken together our studies suggest that the method of FGE-HIPS fluorophore conjugation is predictable, efficient, specific, stable and generally low cost compared to other fluorophore-labeling schemes.

Previous studies suggested that one might separate unlabeled from fluorophore-labeled protein using hydrophobic interaction chromatography^1^. We examined several hydrophobic chromatography matrices including Butyl-S Sepharose, Butyl Sepharose, Phenyl Sepharose or TSKgel Phenyl-5PW. However, none of those approaches separated the highly specific fluorophore-labeled EcMutS from unlabeled protein (Fig. S10). We consider the possibility that hydrophobic chromatography utility may be linked to the solution exposure and/or hydrophobicity of the fluorophore^1^. Nevertheless, a labeling efficiency for proteins that approaches 50% is sufficient for most bulk FRET and single molecule studies.

We determined that the mismatch binding activity of EcMutS-his_6_/ald_6_ during FGE-HIPS labeling and purification was similar and mismatch specific using electrophoretic mobility shift analysis (EMSA; Fig. S11). Real-time bulk kinetic analysis using Surface Plasmon Resonance (SPR; Biacore) revealed minor variations in k_on_, k_off_ and *K*_*D*_ between fluorophore-labeled and unlabeled EcMutS-his_6_/ald_6_ or EcMutS-his_6_/ald_6_(D835R,R840E) that were within the standard error of the system (Table 1; Fig. S12). We also examined the ability of EcMutS-his_6_/ald_6_ to form an ATP-bound sliding clamp by determining the k_off•ATP_ kinetics (Table 1). In all cases the rate appeared similar with the exception of the AF647-labeled EcMutS-his_6_/ald_6_, which appeared to form a sliding clamp approximately 2-fold better than the unlabeled EcMutS-his_6_/ald_6_. All the kinetic rate constants of EcMutS-his_6_/ald_6_ binding and dissociation reported here are similar to the values obtained for EcMutS (without ald_6_-tag) in previous studies^32^,^47^.

To demonstrate utility for *in singulo studies, p*rism-based total internal reflection fluorescence (TIRF) microscopy was used to image single molecules of AF647-labeled EcMutS-his_6_/ald_6_ and AF555-labled EcMutS-his_6_/ald_6_(D835R,R840E) on a 17 Kb *λ* DNA containing a single mismatch (Fig. S13A,B). We observed many stable ATP-bound EcMutS sliding clamps that freely diffused along the entire length of the DNA (Fig. S13A,B)^19^,^48^. The 1-dimensional (1D) random walk particle diffusion characteristic was clearly visible and a diffusion coefficient for AF555-labled EcMutS-his_6_/ald_6_(D835R,R840E) (D = 0.044 μm^2^/sec ± 0.014 μm^2^/sec, N = 77) was easily calculated (Fig. S13A,B; Suppl. Movie-1 and Movie-2, respectively). While these are the first images of ATP-bound EcMutS sliding clamp diffusion on mismatched DNA, the observations appear similar to previous single molecule analysis of TaMutS and Sacchromyces cerevisae ScMsh2-ScMsh6^19^,^20^,^48^. These studies demonstrate that HIPS fluorophore-labeled EcMutS is fully functional for multiple known MSH protein activities. Taken as a whole, our studies demonstrate the general applicability of the HIPS fluorophore-labeling method in bulk and single molecule fluorescence-based analysis.

## Discussion

Although some proteins tagged with an FGE recognition sequence contain converted fGly following co-expression of the FGE protein *in vivo*, this appears not to be the case for all proteins and definitely not with the *E.coli* MMR proteins. While the factors that allow significant conversion *in vivo* are not entirely clear, it appears that the ratio and distribution of soluble FGE and ald_6_-tagged protein are substantial contributors to conversion efficiency. Our results indicate that high expression of FGE may reduce the expression of an ald_6_-tagged protein in *E.coli*, while low expression of FGE can lead to incomplete fGly conversion and reduced labeling efficiency. Moreover, many cellular enzymes exist that may catalyze the modification of aldehydes *in vivo* rendering them unreactive^38^,^39^,^40^. We have found that the conversion of an ald_6_-tag to fGly *in vitro* is easily managed and may be used with partially purified proteins under conditions where unwanted post-conversion aldehyde products may be significantly reduced.

The development of a His_6_-tagged FGE that is easily overexpressed in *E.coli* and may be enriched in a single Ni-NTA chromatography step to >90% purity makes conversion *in vitro* extremely attractive. In addition we have constructed a His_6_-tagged FGE containing a human *Rinovirus* (HRV) 3C protease site capable of removing the his_6_-tag at 0 °C. This latter construct allows conversion *in vitro* and HIPS fluorophore labeling in the presence of HRV 3C protease that may then be followed by Ni-NTA chromatography, which will remove both unincorporated HIPS-fluorophore and the FGE catalytic protein.

The dramatically reduced concentrations of HIPS modified fluorophores required for protein labeling makes this method significantly more cost effective than previous approaches^26^. In fact, the FGE-conversion *in vitro* and HIPS-fluorophore labeling (FGE-HIPS) system appears comparable in efficiency to maleimide-based Cys residue chemical labeling that we have previously used with TaMutS^19^. With the advent of commercially available HIPS-fluorophores, this technology should be widely useful to the scientific community. A major limitation to increasing labeling efficiency is the requirement that the MMR proteins must be maintained at 0 °C in order to preserve specific activity. However, for proteins that maintain activity at elevated temperatures labeling efficiency may be dramatically increased such that at 20–37 °C saturated labeling may occur in a matter of hours (Fig. 2A). In general, we find that labeling efficiency may be increased with higher concentration of fluorophore, longer labeling times, and elevated temperature. We also note that only site-specific labeling increases with longer labeling times (Fig. S5). However, in spite of 99:1 fGly:Cys conversion ratio the site-specific HIPS labeling reaction saturates at ~80%. It should be noted that this saturation efficiency is comparable to virtually all the current fluorophore labeling technologies and likely reflects labeling equilibrium dynamics. In conclusion, we have described an FGE-based fluorophore-labeling method that uses an fGly conversion step *in vitro* followed by Hydrazinyl-Iso-Pictet-Spengler ligation under mild solution conditions. The method displays high specificity with little, if any, effect on protein activity. The specificity of the FGE recognition sequence and relatively low cost of this method makes it generally useful for bulk and single molecule imaging studies that rely on fluorophore-labeling of component proteins.

## Methods

MMR genes were amplified by PCR with primers containing ald_6_ (LCTPSR) tags and inserted into expression plasmids. Proteins were then purified from *E. coli.* stains with the plasmids. Detail information are described in the Supplementary material.

MMR proteins containing an ald_6_-tag were converted with MtFGE *in vitro* and then changed into labeling buffer. HiPS dyes were then added to label the proteins. Detail information are described in the Supplementary material.

SPR experiments were performed as previously described^49^ and the detail information are described in the Supplementary material.

A single molecule Fluorophore Tracking (smFT) apparatus constructed with prism-type Total Internal Reflection Fluorescence (TIRF) microscopy as described^48^. A 17 kb DNA with a single mismatch located 6 Kb from one end was constructed similar to our previous publication^48^. Detail information are described in the Supplementary material.

## Additional Information

**How to cite this article**: Liu, J. *et al.* An Efficient Site-Specific Method for Irreversible Covalent Labeling of Proteins with a Fluorophore. *Sci. Rep.* **5**, 16883; doi: 10.1038/srep16883 (2015).

## Supplementary Material

Supplementary Information

Supplementary Movie-1

Supplementary Movie-2

## Figures and Tables

**Table 1:** DNA Binding, Dissociation and ATP Processing Constants for *E.coli* MutS.

Protein	*k*_*on*_ (10^5^ × M^−1^ × sec^−1^)	*k*_*off*_ (10^−4^ × sec^−1^)	*K*_*D*_ (nM)	*k*_*off·ATP*_ (sec^−1^)
AF555 labeled EcMutS- his_6_/ald_6_(D835R, R840E)	4.37 ± 0.90	24.48 ± 1.44	5.75 ± 1.51	0.45 ± 0.09
Unlabeled EcMutS- his_6_/ald_6_ (D835R, R840E)	9.48 ± 3.90	33.02 ± 1.87	3.85 ± 1.78	0.48 ± 0.09
AF647 labeled EcMutS- his_6_/ald_6_	3.44 ± 0.52	8.93 ± 0.34	2.63 ± 0.49	0.26 ± 0.08
Unlabeled EcMutS- his_6_/ald_6_	5.29 ± 1.82	6.10 ± 0.53	1.25 ± 0.53	0.53 ± 0.11

## References

[b1] Shi X. *et al.* Quantitative fluorescence labeling of aldehyde-tagged proteins for single-molecule imaging. Nat Methods 9, 499–503 (2012). 22466795 10.1038/nmeth.1954 PMC3445270

[b2] Selvin P. R. & Ha T. Single Molecule Techniques: a laboratory manual, (CSHL Press, Cold Spring Harbor, N.Y. 2008).

[b3] Abbondanzieri E. A. *et al.* Dynamic binding orientations direct activity of HIV reverse transcriptase. Nature 453, 184–9 (2008). 18464735 10.1038/nature06941 PMC2655135

[b4] Liu S., Abbondanzieri E. A., Rausch J. W., Le Grice S. F. & Zhuang X. Slide into action: dynamic shuttling of HIV reverse transcriptase on nucleic acid substrates. Science 322, 1092–7 (2008). 19008444 10.1126/science.1163108 PMC2717043

[b5] Lee W., Jose D., Phelps C., Marcus A. H. & von Hippel P. H. A Single-Molecule View of the Assembly Pathway, Subunit Stoichiometry, and Unwinding Activity of the Bacteriophage T4 Primosome (helicase-primase) Complex. Biochemistry 52, 3157–70 (2013). 23578280 10.1021/bi400231s PMC3665360

[b6] Ibarra B. *et al.* Proofreading dynamics of a processive DNA polymerase. EMBO J 28, 2794–802 (2009). 19661923 10.1038/emboj.2009.219 PMC2750014

[b7] Manosas M., Perumal S. K., Croquette V. & Benkovic S. J. Direct observation of stalled fork restart via fork regression in the T4 replication system. Science 338, 1217–20 (2012). 23197534 10.1126/science.1225437 PMC3858903

[b8] Mukhopadhyay J. *et al.* Fluorescence resonance energy transfer (FRET) in analysis of transcription-complex structure and function. Methods Enzymol 371, 144–59 (2003). 14712697 10.1016/S0076-6879(03)71010-6

[b9] Coban O., Lamb D. C., Zaychikov E., Heumann H. & Nienhaus G. U. Conformational heterogeneity in RNA polymerase observed by single-pair FRET microscopy. Biophys J 90, 4605–17 (2006). 16581837 10.1529/biophysj.105.078840 PMC1471840

[b10] Kapanidis A. N. *et al.* Initial transcription by RNA polymerase proceeds through a DNA-scrunching mechanism. Science 314, 1144–7 (2006). 17110578 10.1126/science.1131399 PMC2754788

[b11] Chakraborty A. *et al.* Opening and closing of the bacterial RNA polymerase clamp. Science 337, 591–5 (2012). 22859489 10.1126/science.1218716 PMC3626110

[b12] Davenport R. J., Wuite G. J., Landick R. & Bustamante C. Single-molecule study of transcriptional pausing and arrest by E. coli RNA polymerase. Science 287, 2497–500 (2000). 10741971 10.1126/science.287.5462.2497

[b13] Sakata-Sogawa K. & Shimamoto N. RNA polymerase can track a DNA groove during promoter search. Proc Natl Acad Sci USA 101, 14731–5 (2004). 15469913 10.1073/pnas.0406441101 PMC522051

[b14] Harada Y. *et al.* Direct observation of DNA rotation during transcription by Escherichia coli RNA polymerase. Nature 409, 113–5 (2001). 11343125 10.1038/35051126

[b15] Blanchard S. C., Gonzalez R. L., Kim H. D., Chu S. & Puglisi J. D. tRNA selection and kinetic proofreading in translation. Nat Struct Mol Biol 11, 1008–14 (2004). 15448679 10.1038/nsmb831

[b16] Uemura S. *et al.* Real-time tRNA transit on single translating ribosomes at codon resolution. Nature 464, 1012–7 (2010). 20393556 10.1038/nature08925 PMC4466108

[b17] Qu X. *et al.* The ribosome uses two active mechanisms to unwind messenger RNA during translation. Nature 475, 118–21 (2011). 21734708 10.1038/nature10126 PMC4170678

[b18] Blainey P. C., van Oijen A. M., Banerjee A., Verdine G. L. & Xie X. S. A base-excision DNA-repair protein finds intrahelical lesion bases by fast sliding in contact with DNA. Proc Natl Acad Sci USA 103, 5752–7 (2006). 16585517 10.1073/pnas.0509723103 PMC1458645

[b19] Jeong C. *et al.* MutS switches between two fundamentally distinct clamps during mismatch repair. Nat Struct Mol Biol 18, 379–85 (2011). 21278758 10.1038/nsmb.2009 PMC3060787

[b20] Gorman J. *et al.* Single-molecule imaging reveals target-search mechanisms during DNA mismatch repair. Proc Natl Acad Sci USA 109, E3074–83 (2012). 23012240 10.1073/pnas.1211364109 PMC3494904

[b21] Qiu R. *et al.* Large conformational changes in MutS during DNA scanning, mismatch recognition and repair signalling. Embo J 31, 2528–40 (2012). 22505031 10.1038/emboj.2012.95 PMC3365432

[b22] Howan K. *et al.* Initiation of transcription-coupled repair characterized at single-molecule resolution. Nature 490, 431–4 (2012). 22960746 10.1038/nature11430 PMC3475728

[b23] Resch-Genger U., Grabolle M., Cavaliere-Jaricot S., Nitschke R. & Nann T. Quantum dots versus organic dyes as fluorescent labels. Nat Methods 5, 763–75 (2008). 18756197 10.1038/nmeth.1248

[b24] Hanne J., Liu J., Lee J.-B. & Fishel R. Single-molecule FRET Studies on DNA Mismatch Repair. International Journal of Biophysics 3, 18–38 (2013).

[b25] Roeser D. *et al.* A general binding mechanism for all human sulfatases by the formylglycine-generating enzyme. Proc Natl Acad Sci USA 103, 81–6 (2006). 16368756 10.1073/pnas.0507592102 PMC1324989

[b26] Carrico I. S., Carlson B. L. & Bertozzi C. R. Introducing genetically encoded aldehydes into proteins. Nat Chem Biol 3, 321–2 (2007). 17450134 10.1038/nchembio878

[b27] Rush J. S. & Bertozzi C. R. New aldehyde tag sequences identified by screening formylglycine generating enzymes in vitro and in vivo. J Am Chem Soc 130, 12240–1 (2008). 18722427 10.1021/ja804530w PMC2721638

[b28] Friedberg E. C. *et al.* DNA Repair and Mutagenesis, (American Society of Microbiology, Washington, D.C., 2006).

[b29] Fishel R. & Wilson T. MutS homologs in mammalian cells. [Review] [84 refs]. Curr Opin Genet Dev 7, 105–13 (1997). 9024626 10.1016/s0959-437x(97)80117-7

[b30] Kolodner R. Biochemistry and genetics of eukaryotic mismatch repair. [Review] [85 refs]. Genes & Development 10, 1433–42 (1996). 8666228 10.1101/gad.10.12.1433

[b31] Kolodner R. D., Mendillo M. L. & Putnam C. D. Coupling distant sites in DNA during DNA mismatch repair. Proc Natl Acad Sci USA 104, 12953–4 (2007). 17664420 10.1073/pnas.0705698104 PMC1941800

[b32] Acharya S., Foster P. L., Brooks P. & Fishel R. The coordinated functions of the E. coli MutS and MutL proteins in mismatch repair. Molecular Cell. 12, 233–46 (2003). 12887908 10.1016/s1097-2765(03)00219-3

[b33] Dirksen A. & Dawson P. E. Rapid oxime and hydrazone ligations with aromatic aldehydes for biomolecular labeling. Bioconjug Chem 19, 2543–8 (2008). 19053314 10.1021/bc800310p PMC2761707

[b34] Kalia J. & Raines R. T. Hydrolytic stability of hydrazones and oximes. Angew Chem Int Ed Engl 47, 7523–6 (2008). 18712739 10.1002/anie.200802651 PMC2743602

[b35] Agarwal P. *et al.* Hydrazino-Pictet-Spengler ligation as a biocompatible method for the generation of stable protein conjugates. Bioconjug Chem 24, 846–51 (2013). 23731037 10.1021/bc400042a

[b36] Agarwal P., van der Weijden J., Sletten E. M., Rabuka D. & Bertozzi C. R. A Pictet-Spengler ligation for protein chemical modification. Proc Natl Acad Sci USA 110, 46–51 (2013). 23237853 10.1073/pnas.1213186110 PMC3538270

[b37] Wu P. *et al.* Site-specific chemical modification of recombinant proteins produced in mammalian cells by using the genetically encoded aldehyde tag. Proc Natl Acad Sci USA 106, 3000–5 (2009). 19202059 10.1073/pnas.0807820106 PMC2651276

[b38] Vasiliou V., Pappa A. & Petersen D. R. Role of aldehyde dehydrogenases in endogenous and xenobiotic metabolism. Chem Biol Interact 129, 1–19 (2000). 11154732 10.1016/s0009-2797(00)00211-8

[b39] Sophos N. A. & Vasiliou V. Aldehyde dehydrogenase gene superfamily: the 2002 update. Chem Biol Interact 143–144, 5–22 (2003). 10.1016/s0009-2797(02)00163-1 12604184

[b40] Ho K. K. & Weiner H. Isolation and characterization of an aldehyde dehydrogenase encoded by the aldB gene of Escherichia coli. J Bacteriol 187, 1067–73 (2005). 15659684 10.1128/JB.187.3.1067-1073.2005 PMC545698

[b41] Carlson B. L. *et al.* Function and structure of a prokaryotic formylglycine-generating enzyme. J Biol Chem 283, 20117–25 (2008). 18390551 10.1074/jbc.M800217200 PMC2459300

[b42] Holder P. G. *et al.* Reconstitution of Formylglycine-generating Enzyme with Copper(II) for Aldehyde Tag Conversion. J Biol Chem 290, 15730–45 (2015). 25931126 10.1074/jbc.M115.652669 PMC4505483

[b43] Bojarova P. & Williams S. J. Sulfotransferases, sulfatases and formylglycine-generating enzymes: a sulfation fascination. Curr Opin Chem Biol 12, 573–81 (2008). 18625336 10.1016/j.cbpa.2008.06.018

[b44] Grilley M., Welsh K. M., Su S. S. & Modrich P. Isolation and characterization of the Escherichia coli mutL gene product. J Biol Chem 264, 1000–4 (1989). 2536011

[b45] Su S.-S. & Modrich P. Escherichia coli mutS-encoded protein binds to mismatched DNA base pairs. Proc. Natl. Acad. Sci. USA 83, 5057–5061 (1986). 3014530 10.1073/pnas.83.14.5057 PMC323889

[b46] Mendillo M. L., Putnam C. D. & Kolodner R. D. Escherichia coli MutS tetramerization domain structure reveals that stable dimers but not tetramers are essential for DNA mismatch repair in vivo. J Biol Chem 282, 16345–54 (2007). 17426027 10.1074/jbc.M700858200

[b47] Martin-Lopez J. *et al.* The hMSH2(M688R) Lynch Syndrome Mutation may Function as a Dominant Negative. Carcinogenesis 33, 1647–1654 (2012). 22739024 10.1093/carcin/bgs199 PMC3514906

[b48] Cho W. K. *et al.* ATP Alters the Diffusion Mechanics of MutS on Mismatched DNA. Structure 20, 1264–74 (2012). 22682745 10.1016/j.str.2012.04.017 PMC3974879

[b49] Mazurek A., Johnson C. N., Germann M. W. & Fishel R. Sequence context effect for hMSH2-hMSH6 mismatch-dependent activation. Proc Natl Acad Sci USA 106, 4177–82 (2009). 19237577 10.1073/pnas.0808572106 PMC2657375

