# Supplementary material for: An Efficient Site-Specific Method for Irreversible Covalent Labeling of Proteins with a Fluorophore
Source: Sci Rep. 2015 Nov 19;5:16883. doi: 10.1038/srep16883 (PMC4652282; doi:10.1038/srep16883)
Supplement: Supplementary Information [file srep16883-s1.pdf]

SUPPLEMENTARY MATERIAL

An Efficient Site-Specific Method for Irreversible Covalent Labeling of Proteins with a Fluorophore

Jiaquan Liu[1], Jeungphill Hanne[1], Brooke Britton[1], Aaron E. Albers[2], Matthew Shoffner[1], Jared Bennett[1], Rachel Zatezalo[1], Robyn Barfield[2], David Rabuka[2], Jong-Bong Lee[3, 4] and Richard Fishel[1, 5, *]

[1]Department of Molecular Virology, Immunology and Medical Genetics, The Ohio State University Medical Center, Columbus, OH 43210

[2]Redwood Bioscience, Emeryville, CA 94608

[3]Department of Physics, Pohang University of Science and Technology (POSTECH), Pohang, Korea

[4]School of Interdisciplinary Bioscience and Bioengineering, POSTECH, Pohang, Korea

[5]Physics Department, The Ohio State University, Columbus, OH 43210

*To whom correspondence should be addressed

Additional Methods

Construction of the EcMutS-his_6/ald_6, EcMutL-his_6/ald_6, EcRecJ-his_6/ald_6 and his_6-MtFGE expression plasmids. The *E.coli* EcMutS gene was amplified by PCR with primers containing C-terminal his_6 and ald_6 (LCTPSR) tags (Table 1), digested with XbaI and BamHI, and inserted into pET29a for bacterial expression. The resulting EcMutS-his_6/ald_6 expression plasmid was amplified in *E.coli* XL10 gold and verified by complete DNA sequencing. The EcMutS-his_6/ald_6(C865A) expression plasmid was constructed similarly except with a different C-terminal primer (Table 1). EcMutS-his_6/ald_6(D835R,R840E) expression plasmid was generated using QuikChange site-directed mutagenesis (Stratagene) from the EcMutS-his_6/ald_6 expression plasmid. *E. coli.* EcMutL gene was amplified by PCR with primers containing C-terminal his_6 and ald_6 tags (Table 1), digested with Nde I and Xho I, and inserted into pET29a bacterial expression plasmid. The resulting construct was amplified in *E. coli.* XL 10 gold strain and verified by complete DNA sequencing. EcMutL(346 ald_6)-his_6 expression plasmid was generated using QuikChange site-directed mutagenesis (Stratagene) from the EcMutL-his_6/ald_6 expression plasmid *E. coli.* RecJ gene was amplified by PCR with primers containing C-terminal ald_6 and his_6 tags (Table 1), digested with XbaI and BamHI, and inserted into pET9a bacterial expression plasmid. The resulting construct was amplified in *E. coli.* XL 10 gold strain and verified by complete DNA sequencing. *Mycobacterium tuberculosis* MtFGE gene was amplified by PCR with primers (Table 1), digested with Nhe I and Hind III, and inserted into the pBAD42 bacterial expression plasmid. The resulting construct was amplified in *E. coli*. XL 10 gold strain and verified by complete DNA sequencing. The his_6-MtFGE expression plasmid (in pET14) was obtained from Redwood Bioscience (Emeryville, CA).

EcMutS expression and partial purification. After co-transformation with the MtFGE and EcMutS expression plasmids, BL21 AI cells were cultured at 37°C for 1h, spread on an LB-spectinomycin-kanamycin plate, and grown at 37°C overnight. A single colony was transferred

to 10 ml of LB with 100 µg/mL spectinomycin and 10 µg/ml kanamycin at 37 °C for overnight, followed by 1 L of LB medium containing the same antibiotics. Expression of MtFGE was induced by addition of L-(+)-Arabinose (0.2 % wt/vol) at OD_{600} = 0.3. The growth temperature was decreased to 16°C and EcMutS induced 30 m later by addition of IPTG (0.05 mM). Cells were collected after 16h and resuspended in freezing buffer (25 mM Hepes pH 7.8, 300 mM NaCl, 10 % glycerol and 20 mM imidazole). Cell pellets were frozen-thaw three times and sonicated twice, followed by centrifuged at 41000 rpm (Rotor: Ti 60 Beckman) for 1h. The supernatants were then loaded on a Ni-NTA (Qiagen) column equilibrated in Buffer A (25 mM Hepes pH 7.8, 10 % glycerol and 20 mM imidazole) containing 500 mM NaCl, washed with Buffer A containing 500 mM NaCl followed by Buffer A containing 300 mM NaCl and eluted with 20-200 mM Imidazole step in Buffer A plus 300 mM NaCl.

EcMutL expression and partial purification. After transformation with the EcMutL expression plasmid, BL21 AI cells were cultured at 37°C for 1h, spread on an LB- kanamycin plate, and grown at 37°C overnight. A single colony was transferred to 10 mL of LB containing 10 µg/ml kanamycin at 37°C for overnight, followed by dilution into 1 L of LB containing the same antibiotic. Expression of EcMutL was induced by addition of L-(+)-Arabinose (0.2 % wt/vol) and IPTG (0.2 mM) at OD_{600} = 0.3. Cells were collected after 3h and resuspended in freezing buffer (25 mM Hepes pH 7.8, 300 mM NaCl, 10% glycerol and 20 mM imidazole). Cell pellets were frozen-thaw three times and sonicated twice, followed by centrifuged at 41000 rpm (Rotor: Ti 60 Beckman) for 1h. The supernatants were then loaded on a Ni-NTA (Qiagen) column equilibrated in Buffer A (25 mM Hepes pH 7.8, 10 % glycerol and 20 mM imidazole) containing 500 mM NaCl, washed with Buffer A containing 500 mM NaCl followed by Buffer A containing 300 mM NaCl and eluted with a 20-200 mM Imidazole step in Buffer A plus 300 mM NaCl.

EcRecJ expression and partial purification. After transformation with the EcRecJ expression plasmid, BL21 AI cells were cultured at 37°C for 1h, spread on an LB- kanamycin plate, and grown at 37°C overnight. A single colony was transferred to 10 mL of LB containing 10 µg/ml kanamycin at 37°C for overnight, followed by dilution into 1 L of LB containing the same antibiotic. At OD_{600} = 0.3 the growth temperature was decreased to 16°C and expression of EcRecJ was induced by addition of L-(+)-Arabinose (0.2% wt/vol) and IPTG (0.2 mM). Cells were collected after 16h and resuspended in freezing buffer (25 mM Hepes pH 7.8, 300 mM NaCl, 10% glycerol and 20 mM imidazole). Cell pellets were frozen-thaw three times and sonicated twice, followed by centrifuged at 41000 rpm (Rotor: Ti 60 Beckman) for 1h. The supernatants were then loaded on a Ni-NTA (Qiagen) column equilibrated in Buffer A (25 mM Hepes pH 7.8, 10 % glycerol and 20 mM imidazole) containing 500 mM NaCl, washed with Buffer A containing 500 mM NaCl followed by Buffer A containing 300 mM NaCl and eluted with a 20-200 mM Imidazole step in Buffer A plus 300 mM NaCl.

MtFGE expression and purification. After transformation with the MtFGE expression plasmid, BL21 cells were cultured at 37°C for 1h, spread on an LB-ampicillin plate, and grown at 37°C overnight. A single colony was transferred to 10 mL of LB containing 50 µg/ml ampicillin at 37°C for overnight, followed by dilution into 1 L of LB containing the same antibiotic. At OD_{600} = 0.3 the growth temperature was decreased to 16°C and expression of MtFGE was induced 30m later by addition of IPTG (0.1 mM). Cells were collected after 16h and resuspended in freezing buffer (25 mM Hepes pH 7.8, 300 mM NaCl, 10% glycerol and 20 mM imidazole). Cells were collected after 16h and resuspended in freezing buffer (25 mM Hepes pH 7.8, 300 mM NaCl, 10 % glycerol and 20 mM imidazole). Cell pellets were frozen-thaw three times and sonicated twice, followed by centrifuged at 41000 rpm (Rotor: Ti 60 Beckman) for 1h. The supernatants were then loaded on a Ni-NTA (Qiagen) column equilibrated in Buffer A (25 mM Hepes pH 7.8, 10 % glycerol and 20 mM imidazole) containing 500 mM NaCl, washed with Buffer A containing 500 mM NaCl

followed by Buffer A containing 150 mM NaCl and eluted with linear 20-200 mM Imidazole gradient in Buffer A plus 150 mM NaCl. MtFGE-containing fractions were dialyzed in storage buffer (25 mM Hepes pH 7.8, 1 mM DTT, 0.1 mM EDTA, 150 mM NaCl, 20 % glycerol) and frozen at -80°C. MtFGE retained full fGly conversion activity *in vitro* after 1y at -80°C.

MtFGE conversion and fluorophore-labeling. MMR proteins containing an ald_6-tag and MtFGE (ratio 1:1) were dialyzed together in conversion buffer (50 mM Tris-HCl pH 8.0, 100 mM NaCl, 20 mM Arginine and 0.25 mM DTT) at 4°C for 48h and then dialyzed in labeling buffer (100 mM potassium-phosphate pH 6.4, 0.25 mM DTT, 300 mM NaCl) for overnight. Proteins were then incubated with AlexaFluor or Atto-HiPS dye at 0°C for 48h.

EcMutS purification after labeling. After labeling, EcMutS was diluted with 3 volume of Buffer B (25 mM Hepes pH 7.8, 1 mM DTT, 10 % glycerol) and loaded onto a heparin column, washed with Buffer B plus 100 mM NaCl and eluted with a linear gradient of 100 mM – 1 M NaCl. Fractions were visualized on an 8% SDS-PAGE, scanned on a typhoon 9410 (GE Healthcare Life Sciences) and then stained with Coomassie Blue. EcMutS-containing fractions were dialyzed in storage buffer (25 mM Hepes pH 7.8, 1 mM DTT, 0.1 mM EDTA, 150 mM NaCl, 20 % glycerol,) and frozen at -80°C.

EcMutL purification after labeling. After labeling, EcMutL was diluted with 10 volume of labeling buffer without DTT and loaded on a Ni-NTA (Qiagen) column equilibrated in Buffer A (25 mM Hepes pH 7.8, 10 % glycerol and 20 mM imidazole) plus 25 mM NaCl, washed with Buffer A containing 25 mM NaCl and eluted directly on to a ssDNA cellulose column (Affimetrix) with a 20-200 mM Imidazole step in Buffer A plus 25 mM NaCl. The ssDNA cellulose column was washed

with Buffer B plus 25 mM NaCl and EcMutL eluted with a 25 mM – 1 M NaCl linear gradient. EcMutL-containing fractions eluting at 80-100 mM NaCl were pooled, diluted with 1 volume Buffer B containing 50 mM NaCl, loaded onto MonoS equilibrated in Buffer B plus 100 mM NaCl, and eluted with a 100 mM – 1 M NaCl linear gradient in Buffer B. Fractions were visualized on an 8% SDS-PAGE, scanned on a typhoon 9410 (GE Healthcare Life Sciences) and then stained with Coomassie Blue. EcMutL-containing fractions were dialyzed in storage buffer (25 mM Hepes pH 7.8, 1 mM DTT, 0.1 mM EDTA, 150 mM NaCl, 20 % glycerol) and frozen at -80°C. A 12% SDS-PAGE gel was used to separate labeled and unlabeled EcMutL and quantification was performed using ImageQuant software.

Gel mobility shift assay. A 41 bp Cy3-labeled DNA with or without a central G/T mismatch (3 nM) was inclubated on ice for 15 m with EcMutS (400 nM) in 20 μL GS Buffer (25 mM Hepes pH 7.8, 1 mM DTT, 2 mM $MgCl_2$, 130 mM NaCl, 15 % glycerol, 100 μg/ml acetylated BSA and 5 ng/μl poly dI·dC). Reactions were immediately loaded and separated on a 5% polyacrylamide (29:1 bis), 4 % glycerol gel in TAE Buffer (40 mM Tris Acetate, 1 mM EDTA). Gels were scanned on typhoon 9410 (GE Healthcare Life Sciences).

Surface plasmon resonance (SPR). Biotinylated DNA (Table 1) was immobilized on the streptavidin coated chip surface by non-covalent capture. Experiments were carried out at 25°C. Binding experiments consisted of five EcMutS dimeric protein concentrations: 0 nM, 10 nM, 20 nM, 50 nM or 100 nM in Reaction Buffer containing 25 mM Hepes pH 7.8, 1 mM DTT, 1 mM $MgCl_2$, 130 mM NaCl, 10 % glycerol, 100 μg/ml acetylated BSA (Promega), 0.005 % P-20 surfactant (BIAcore). ATP-induced dissociation was followed in Reaction Buffer containing 0.5 mM ATP following EcMutS binding.

Hydrophobic interaction chromatography. Fluorophore-labeled EcMutS-his$_6$/ald$_6$ was diluted into 20 volumes of Buffer C (50 mM sodium phosphate pH 8.0, 1 mM DTT, 0.1 mM EDTA, 10 % glycerol) plus 0.6 M K_2HPO_4, loaded onto a Butyl Sepharose 4 Fast Flow column (GE Healthcare Life Sciences) equilibrated in Buffer C plus 0.6 M K_2HPO_4 and eluted with a linear gradient from 0.6 M – 0 M K_2HPO_4 in Buffer C).

Protein concentration, dye concentration and labeling efficiency calculation. The concentrations of unlabeled proteins were determined by measuring absorbance at 280 nm (EcMutS, ε_{280} = 73,230 cm^{-1}M^{-1}, EcMutL ε_{280} = 54,890 cm^{-1}M^{-1}). The concentrations of AF dyes were determined by measuring absorbance at 555 nm (AF555, ε_{555} = 150,000 cm^{-1}M^{-1}) or 650 nm (AF647, ε_{650} = 239,000 cm^{-1}M^{-1}). The labeling ratios of proteins were determined by measuring protein absorbance and dye absorbance, respectively. The concentration of labeled protein was corrected for the absorbance of the dyes at 280 nm (8% of absorbance at 555 nm for AF555 and 3% of absorbance at 650 nm for AF647). The fluorescent image and the coomassie image of each lane on the SDS-PAGE gel were quantified by Image Quant (Molecular Dynamics). For Figure 2, the labeling efficiency of a known reference lane ($Ref_{\%}$) was determined as the ratio of the top band intensity divided by the total intensity of both bands in the coomassie gel and multiple by 100 (Reference lanes in Panel A top: 0.1 mM = 68%; Panel A bottom: 10 mM = 55%; Panel B: 96hr = 54%). The fluorescent intensity (I_F) and the coomassie intensity (I_c) of each lane was quantified by Image Quant (Molecular Dynamics) and the ratio (R) was calculated by:

$$R = \frac{I_F}{I_C}$$

Equation 1

The labeling efficiency of the unknown lanes ($Unk_{\%}$) was then calculated using Equation 2:

$$Unk_{\%} = \frac{R_{unk}}{R_{Ref}} \times Ref_{\%}$$

Equation 2

For Figure 3, Figures 4, Figure S5 and Figure S8, The lanes with highest ratio (R, see above) were used as references and labeling efficiency were set to 100%, the Relative Labeling Efficiency was calculated using Equation 3.

$$Unk_{\%} = \frac{R_{unk}}{R_{Ref}} \times 100$$

Equation 3

Single molecule tracking of EcMutS on lambda DNA containing a single mismatch. A 17kb DNA with a single mismatch located 6 Kb from one end was constructed. A 30-mer oligonucleotide containing a 5'-biotin was ligated to the two ends of the 17 Kb DNA followed by injection into a custom-made flow cell. The 17 Kb DNA was stretched by laminar flow (200 μl/min) and grafted onto a neutravidin coated quartz slide passivated surface. Unbound DNA was removed with gentle flow (10 μl/min) to prevent disruption of stretched DNA and the flow cell equilibrated in T50 Buffer (10mM Tris-HCl pH 7.5, 50mM NaCl). AF555-labeled EcMutS and when indicated AF647-labeled EcMutL in imaging buffer plus 1 mM ATP was introduced by the slow laminar flow (10 μl/min). The syringe pump was then clamped closed and protein-DNA interactions were monitored with an EM CCD camera with appropriate excitation lasers. Single molecule tracking was performed using an in-house modified version of the DiaTrack software (Semasopht, North Epping, AU).

Bacterial Strains. The strains used in this study are prototrophic derivatives of the *Escherichia coli* K12 reference strain MG1655, kindly supplied by Patricia L. Foster, Department of Biology, Indiana University, Bloomington, IN 47405. Detail strains information are provided below:

Wild type = MG1655, *rph*+, lambda-, F'-

ΔEcmutS = MG1655, *rph*+, *ΔmutS*, lambda-, F'-

ΔEcmutL = MG1655, *rph*+, *ΔmutL*, lambda-, F'-

***In vivo* complementary drop assay.** Colonies from different strains without a plasmid were inoculated into 5 ml of VB Minimal LGT Media (Vogel-Bonner + Glucose + Thiamine) and culture overnight. Colonies from different strains with plasmids (co-transform with pTARA (addgene 31491) and plasmid of interest) were inoculated into 5 ml of VB Minimal LGT Media (Vogel-Bonner + Glucose + Thiamine) containing 50 µg/ml Kanamycin, 35 µg/ml Chloramphenicol, 0.2% Arabinose, and IPTG (0.05mM for EcMutS and 0.2 mM for EcMutL) and culture overnight until confluent. Cultures then were diluted 0 - 125 folds and 10 µl drops were plated onto LB Broth (Miller) supplemented with 100 µg/ml rifampicin. Plates were grown at 37°C overnight and imaged.

HiPS Linker Addition. *Chemicals* - AlexaFluor (AF) Cadaverines (488, 555, and 647) were purchased from Life Technologies (Carlsbad, CA). ATTO488 NHS ester was purchased from Sigma-Aldrich (St. Louis, MO). All other chemicals were purchased from Sigma-Aldrich (St. Louis, MO) or VWR (Radnor, PA). All reactions were performed under a dry nitrogen atmosphere unless otherwise indicated. Reversed phase flash column chromatography was performed on a Biotage Isolera One flash purification system using Biotage SNAP KP C18 HS column cartridges fitted with appropriately sized Biotage SNAP KP C18 HS samplet cartridges. HPLC solvents (H_2O and CH_3CN, containing 0.1% v/v HCO_2H) were purchased from VWR (Radnor, PA). Elim Biopharmaceuticals Incorporated (Hayward, CA) performed Electrospray ionization (ESI) low-resolution mass spectral (LRMS) analysis of fluorophore conjugates.

Fluorophore HPLC Analyses. HPLC analyses were conducted on an Agilent 1100 Series Analytical HPLC equipped with a Model G1322A Degasser, Model G1311A Quarternary Pump, Model G1329A Autosampler, Model G1314 Variable Wavelength Detector, and Model G1364C Fraction Collector at room temperature according to the methods described below. Detection was performed at 205 nm, 254 nm, 488 nm (AF488), 495 nm (ATTO488), 555 nm (AF555), and 600 nm (AF647).

METHOD A

Agilent Poroshell 120 SB C18, 4.6 mm × 150 mm (2.7 um) (1.0 mL min^{-1}).

Solvent A: H_2O (0.1% formic acid); Solvent B: Acetonitrile (0.1% formic acid)

Time (min)	Solvent B (%)
0.0	10
15.0	100
17.5	100
18.0	10
20.5	10

METHOD B

Agilent Poroshell 120 SB C18, 4.6 mm × 50 mm (2.7 um) (2.5 mL min^{-1}).

Solvent A: H_2O (0.1% formic acid); Solvent B: Acetonitrile (0.1% formic acid)

Time (min)	Solvent B (%)
0.0	10
5.0	100
6.0	100
6.1	10
7.1	10

Preparation of FGly-Reactive HiPS Indole Cadaverine AlexaFluor Dyes. (Step 1) 9-Fluorenylmethyl Carbamate (FMOC) HiPS Indole Cadaverine AF-dyes was prepared by a modification of our previously described method [1]. To a 4 mL glass scintillation vial containing a flea stir bar was added AF-dye Cadaverine Sodium Salt (15 mg, 0.02 mmol) in H_2O (0.5 mL).

N,N’-Diisopropylethylamine (DIPEA; 18.2 mg, 24.5 mL, 0.14 mmol) was added, followed by slow, dropwise addition of 9-Fluorenylmethyl Carbamate (FMOC) HiPS Indole Pentafluorophenyl Ester (32.7 mg, 0.05 mmol) in N,N’-Dimethylformamide (DMF; 0.5 mL). The reaction was stirred at room temperature for 4 h, adsorbed directly onto a Biotage KP C18 HS 1.2 g samplet, and purified on a Biotage KP C18 HS 12 g cartridge using a gradient of 0-100% acetonitrile (CH_3CN) in H_2O. The FMOC HiPS Indole Cadaverine AF488 was a reddish orange film (24.6 mg, 97% yield) with an ESI-LRMS $C_{55}H_{52}N_7O_{13}S_2{}^{2}$ of 1082.1 compared to the calculated 1082.3.

(Step 2) *Preparation of HiPS Indole Cadaverine AlexaFluor*. To a 4 mL glass scintillation vial containing a flea stir bar was added FMOC HiPS Indole Cadaverine AF-dye from Step 1. Piperidine (38.6 mg, 44.8 mL, 0.5 mmol) in N,N’-Dimethylacetamide (DMA), H_2O (179 mL, 9 mL) was added by syringe. The solution was stirred at room temperature for 20 m, adsorbed directly onto a Biotage KP C18 HS 1.2 g samplet, and purified on a Biotage KP C18 HS 12 g cartridge using a gradient of 0-100% CH_3CN in H_2O. For FMOC HiPS Indole Cadaverine AF488 (24.6 mg, 0.02 mmol) from Step 1 we obtained HIPS Indole Cadaverine AF488 that was a reddish orange film (15.4 mg, 85% yield) with an ESI-LRMS $C_{55}H_{52}N_7O_{13}S_2{}^{2}$ of 860.3 compared to the calculated 860.2. The HIPS Indole Cadaverine AF555 was a purplish red film (15.4 mg, 82% yield); the HIPS Indole Cadaverine AF647 was a dark blue film (15.4 mg, 83% yield).

The molecular structures of the AF555 and AF647 Cadaverine Disodium Salts have not been publicly disclosed by Life Technologies. However, spectroscopic and mass spectral analyses of the cadaverine starting materials, protected dye intermediates, and deprotected final compounds are consistent with HiPS functionalization (data not shown).

Preparation of FGly-Reactive HiPS Indole Cadaverine ATTO Dyes. (Step 1) We first prepared *tert*-Butyl Carbamate (BOC) Cadaverine ATTO-Dye. To a 4 mL glass scintillation vial containing

a dried flea stir bar was added NHS-ATTO-Dye (10 mg, 0.01 mmol), anhydrous DMF (0.3 mL), NHBOC cadaverine (4.1 mg, 4.2 mL, 0.02 mmol), and DIPEA (7.9 mg, 10.6 mL, 0.06 mmol). The reaction was stirred at room temperature for 2 h, adsorbed directly onto a Biotage KP C18 HS 1.2 g samplet, and purified on a Biotage KP C18 HS 12 g cartridge using a gradient of 0-100% CH_3CN in H_2O. The BOC Cadaverine ATTO488 product was a reddish orange film (7.8 mg, 99% yield).

(Step 2) *Preparation of (Trifluoroacetate Salt) Cadaverine ATTO-Dyes*. To a dried 4 mL glass scintillation vial containing a flea stir bar was added BOC Cadaverine ATTO-Dye (~12 mg, 0.02 mmol) and 1 mL of a solution of Trifluoroacetic acid (TFA), Triisopropylsilane (TIPS), H_2O (950 mL, 25 mL, 25 mL, respectively). The reaction was stirred at room temperature for 1 h, evaporated, and dried under high vacuum. The BOC Cadaverine ATTO488 was a red orange film that was used in subsequent reactions without additional purification (12.1 mg, 98% yield).

(Step 3) *Preparation of FMOC HiPS Indole Cadaverine ATTO-Dye*. To a dried 4 mL glass scintillation vial containing a dried flea stir bar was added (Trifluoroacetate Salt) Cadaverine ATTO-Dye (~12 mg, 0.02 mmol) in DMF (200 mL), FMOC HIPS Indole Pentafluorophenyl Ester (21.1 mg, 0.03 mmol), and DIPEA (20.8 mg, 28 mL, 0.2 mmol). The reaction was stirred at room temperature for 2 h, adsorbed directly onto a Biotage KP C18 HS 1.2 g samplet, and purified on a Biotage KP C18 HS 12 g cartridge using a gradient of 0-100% CH_3CN in H_2O. The FMOC HIPS Indole Cadaverine ATTO-488 was an orange film (15.0 mg, 86% yield) with an ESI-LRMS $C_{55}H_{52}N_7O_{13}S_2{}^{2}$ of 1137.1 compared to the calculated 1137.4.

(Step 4) *Preparation of HiPS Indole Cadaverine ATTO-Dye*. To a 4 mL glass scintillation vial containing a flea stir bar was added FMOC HiPS Indole Cadaverine ATTO-Dye (15.0 mg, 0.01 mmol). Piperidine (51.7 mg, 60 mL, 0.6 mmol) in DMA, H_2O (228 mL, 12 mL) was added by syringe. The solution was stirred at room temperature for 20 m, adsorbed directly onto a Biotage

KP C18 HS 1.2 g samplet, and purified on a Biotage KP C18 HS 12 g cartridge using a gradient of 0-100% CH_3CN in H_2O. The HIPS Indole Cadaverine ATTO488 was a reddish orange film (12.0 mg, 99% yield) with an ESI-LRMS $C_{55}H_{52}N_7O_{13}S_2\,^{2}$ of 915.3 compared to the calculated 915.3.

REFERENCES

1. Agarwal, P. et al. Hydrazino-Pictet-Spengler ligation as a biocompatible method for the generation of stable protein conjugates. *Bioconjug Chem* **24**, 846-51 (2013).
2. Szyf, M. et al. DNA methylation pattern is determined by the intracellular level of the methylase. *Proc. Natl. Acad. Sci. U. S. A.* **81**, 3278-3282 (1984).

Table S1. Oligonucleotides used in these studies.

Name	Sequences
MtFGE fwd	GTTCTGTTGGCTAGCAACAGGAGGAATTAACCATGCTGACCGAGTTGGTTGACCT
MtFGE rev	CCAACAGCCAAGCTTCTACCCGGACACCGGGTCGGCCACGCAC
EcMutS-his$_6$/ald$_6$ fwd	TTCCCCTCTAGAAATAATTTTGTTTAACTTTAAGAAGGAGATATACATAATATGAGT GCAATAGAAAATTTCG
EcMutS-his$_6$/ald$_6$ rev	GCCGGATCCTTAACGCGACGGTGTGCAGAGCCCTCCATGATGGTGATGGTGATGTGA TGACACCAGGCTCTTCAAGCGA
EcMutS-his$_6$/ald$_6$ (C865A) rev	GCCGGATCCTTAACGCGACGGTGTTGCGAGCCCTCCATGATGGTGATGGTGATGTGA TGACACCAGGCTCTTCAAGCGA
EcMutS-his$_6$/ald$_6$ (D835R, R840E) fwd	CACTCACCCCGGAACAGGCGCTCGAGTGGATTTATCGCTTGAAGA
EcMutS-his$_6$/ald$_6$ (D835R, R840E) rev	TCTTCAAGCGATAAATCCACTCGAGCGCCTGTTCCGGGGTGAGTG
EcMutS-his$_6$/ald$_6$ (D835R, R840E) G4 rev	GCCGGATCCTTAACGCGACGGTGTGCAGAGCCCCCTATGATGGTGATGGTGATGGCC ACCCCCTCCCACCAGGCTCTTCAAGCGA
EcMutL-his$_6$/ald$_6$ fwd	GGAGGCCATATGCCAATTCAGGTCTTACC
EcMutL-his$_6$/ald$_6$ rev	TCCGCCCTCGAGTTAACGCGACGGTGTGCAGAGCCCTCCATGATGGTGATGGTGATG TGATGACTCATCTTTCAGGGCTTTTATCG
EcMutL(346 ald$_6$)-his$_6$ fwd1	GGAGGCCATATGCCAATTCAGGTCTTACC
EcMutL(346 ald$_6$)-his$_6$ rev1	TCCGCCCTCGAGTTAATGATGGTGATGGTGATGTGATGACTCATCTTTCAGGGCTTT TATCG
EcMutL(346 ald$_6$)-his$_6$ fwd2	CGCTGGACGATGAACCCCAAGGAGGGCTCTGCACACCGTCGCGTGGAGGGGTGGCGG CGGGGCGCAATCA
EcMutL(346 ald$_6$)-his$_6$ rev2	TGATTGCGCCCCGCCGCCACCCCTCCACGCGACGGTGTGCAGAGCCCTCCTTGGGGT TCATCGTCCAGCG
EcRecJ-his$_6$/ald$_6$ fwd	GCGCGCTCTAGAAAGGAGATATACATATGAAACAACAGATACAACTTCGTC
EcRecJ-his$_6$/ald$_6$ rev1	GCCTTTGCCATGATGATGATGATGATGGCCAATTGGCCAGATATTGTCGATGA
EcRecJ-his$_6$/ald$_6$ rev2	GCGCGCGGATCCTTAACGGCTCGGGGTGCACAGGCCTTTGCCATGATGATGATGA
41 bp homoduplex	5′-(6-FAM)TCTTAGGATCATCGAGGATCGACGTCGGTGCAATTCAGCGG-3′<br>3′-AGAATCCTAGTAGCTCCTAGCTGCAGCCACGTTAAGTCGCC-5′
41 bp G/T mismatch	5′-(6-FAM)TCTTAGGATCATCGAGGATCGACGTCGGTGCAATTCAGCGG-3′<br>3′-AGAATCCTAGTAGCTCCTAGTTGCAGCCACGTTAAGTCGCC-5′
G/T mismatch DNA (SPR)	AACTATAGGGCGAATTGGGTACCGCTGAATTGCACCGAGCTTGATCCTCGATGATCC TAAGCTAAGCTTCAGCTCCAGCTTT-3′<br>TTGATATCCCGCTTAACCCATGGCGACTTAACGTGGCTCGAGCTAGGAGCTACTAGG ATTCGATTCGAAGTCGAGGTCGAAA-5′

Table S2. Labeling efficiency of labeled MutS and MutL

Protein	Monomer with a dye	Dimer without dyes	Dimer with single dye	Dimer with dual dyes
AF647 labeled EcMutS-his_6/ald_6	23%	59%	36%	5%
AF555 labeled EcMutS-his_6/ald_6(D835R, R840E)	34%	44%	44%	12%
AF647 labeled EcMutL-his_6/ald_6	35%	42%	46%	12%

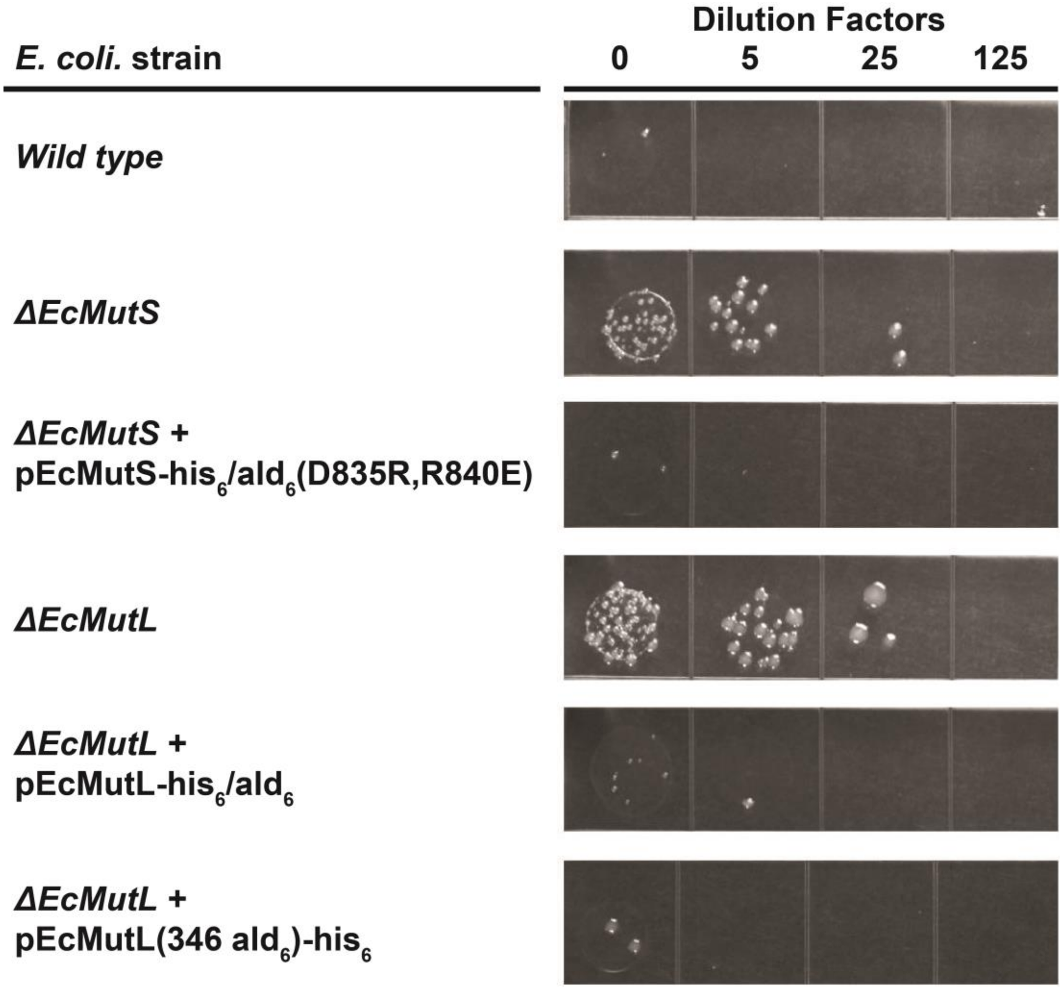

Figure S1. EcMutS or EcMutL with an ald_6 tag is fully functional *in vivo*. Complementation of *E.coli* MG1655 ΔmutS and ΔmutL strains by plasmids expressing EcMutS or EcMutL that contain the FGE recognition sequence (ald_6) and hexa-His (his_6) tag. The relative frequency of rifampicin-resistance (rif^r) was determined by spotting 10 μl dilutions of bacterial culture (shown above) prepared from starting saturated overnight cultures.

A)

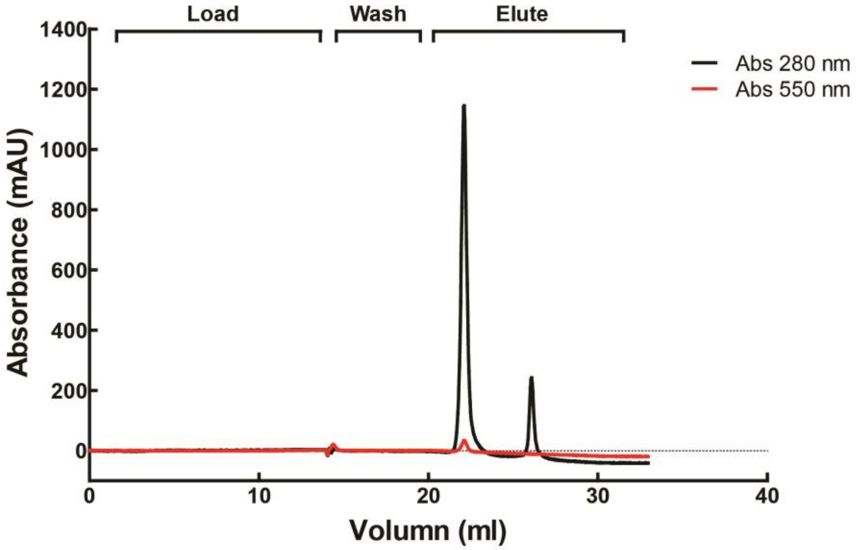

B)

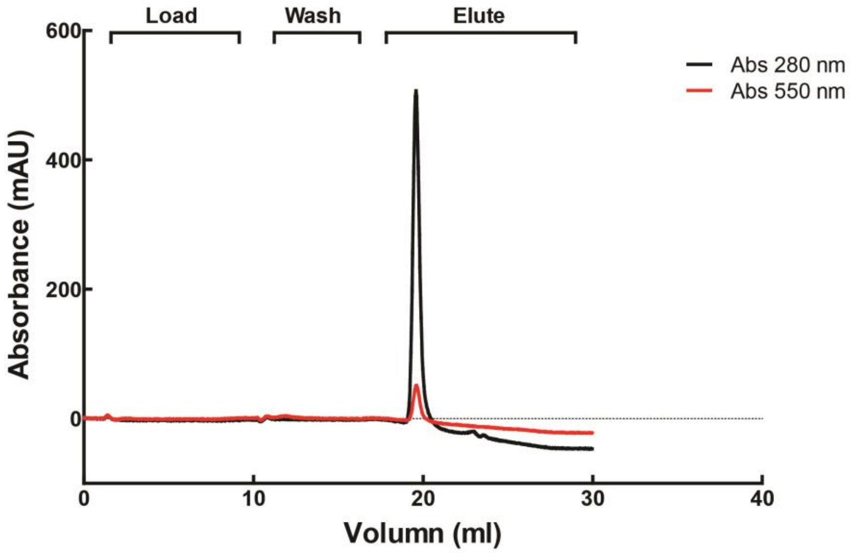

Figure S2. Labeling and purification of EcMutS-his$_6$/ald$_6$ with Cy3-hydrazide. (A) Mono-Q chromatography purification of EcMutS-his$_6$/ald$_6$ labeled with 4 mM Cy3-hydrazide. **(B)** Mono-Q chromatography purification of EcMutS-his$_6$/ald$_6$ labeled with 13 mM Cy3-hydrazide.

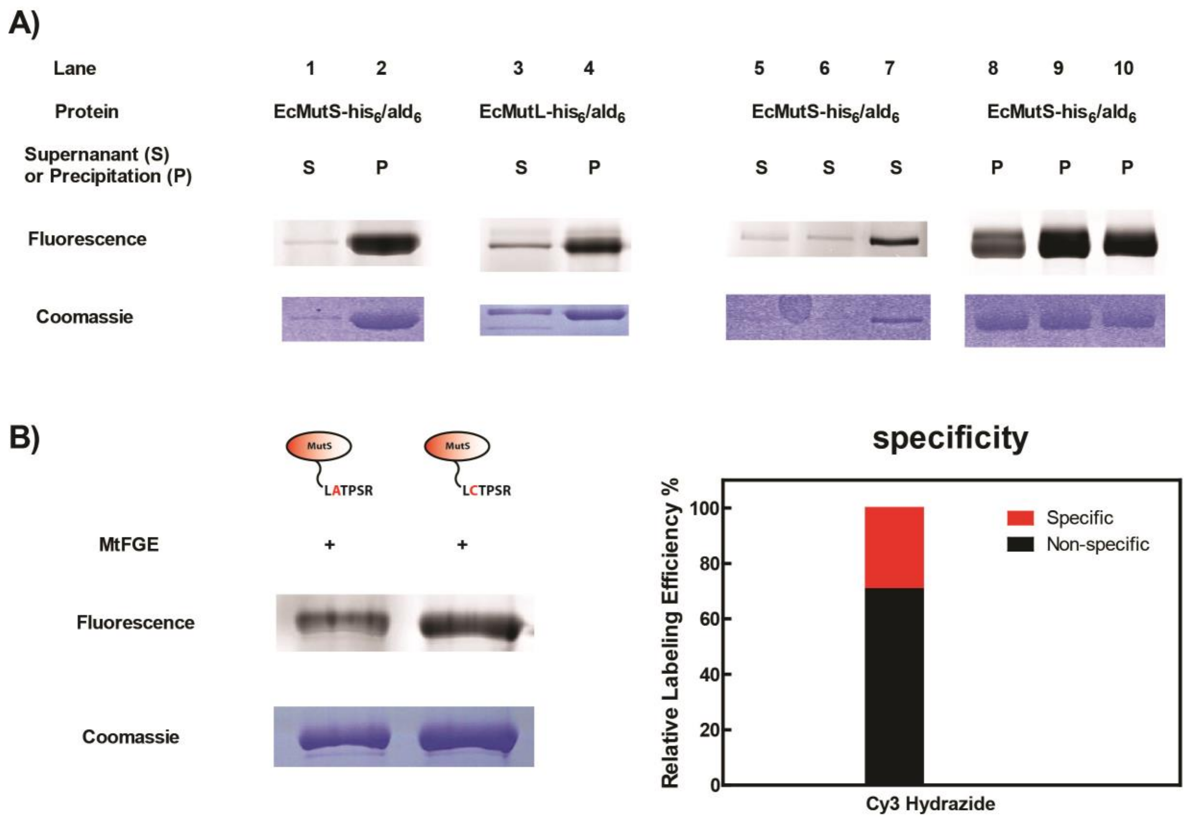

Figure S3. The effect of high Cy3-hydrazide concentration on EcMutS-his$_6$/ald$_6$ and EcMutL-his$_6$/ald$_6$ labeling. (A) High concentration of Cy3-hydrazide precipitated most of EcMutS-his$_6$/ald$_6$ or EcMutL-his$_6$/ald$_6$. Proteins were labeled as described below. Supernatants and precipitations were then separated by centrifuged at 13000 × g. Precipitations (lane 2, 4, 8, 9, 10) were suspended in 0.4 % SDS with the same volume as supernatants (lane 1, 3, 5, 6, 7). Supernatants and precipitations were then loaded on a SDS-PAGE gel to separate free dyes and labeled proteins. EcMutS-his$_6$/ald$_6$ in lane 1, 2 and EcMutL-his$_6$/ald$_6$ in lane 3, 4 were labeled with 66 mM Cy3-hydrazide in 250 mM potassium phosphate pH 7.0, 500 mM KCl and 5 mM DTT; EcMutS-his$_6$/ald$_6$ in lane 5, 8 were labeled in the same condition as lane 1 except with additional 0.01% Tween 20; EcMutS-his$_6$/ald$_6$ in lane 6, 9 were labeled in the same condition as lane 1 except with 150 mM NaCl instead of 500 mM KCl; EcMutS-his$_6$/ald$_6$ in lane 7, 10 were labeled in the same condition as lane 1 except in pH 8.0. **(B)** Fluorescence, coomassie gels and specificity of labeling EcMutS-his$_6$/ald$_6$ with 66 mM Cy3-hydrazide in the same condition as lane 1, 2 in (A) except supernatants and precipitations were not separated but loaded together. Proteins and relative labeling efficiency were calculated as described in Fig. 4.

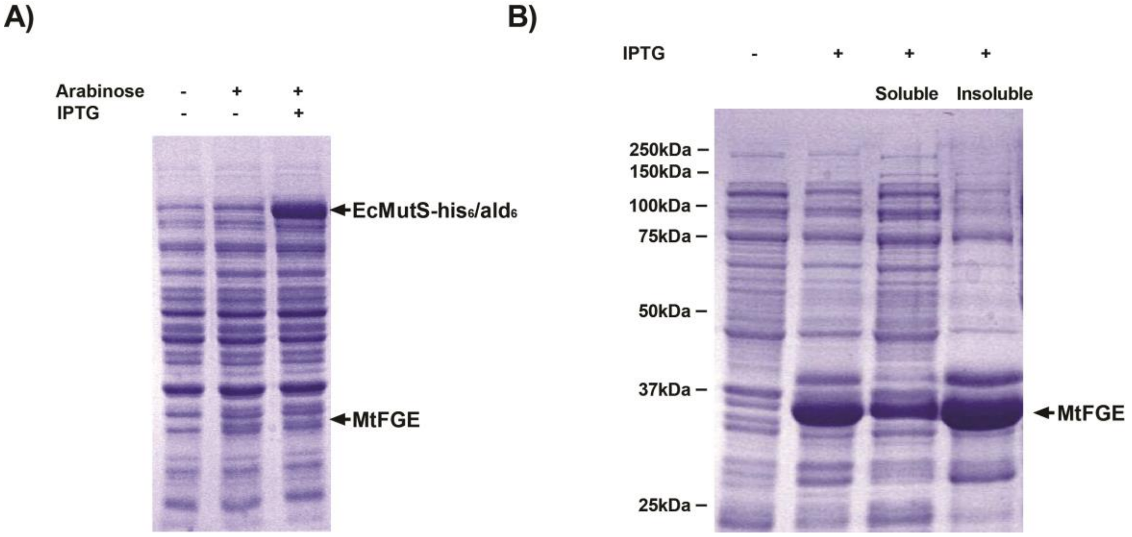

Figure S4. Expression of MtFGE in *E. coli*. (A) Total proteins of EcMutS-his$_6$/ald$_6$ and MtFGE co-expression in BL21 AI strain. **(B)** Total proteins (First and second lanes from left) and soluble/insoluble proteins of MtFGE expression in BL21 (DE3) strain. Cell pellets were sonicated twice, followed by centrifuged at 13000 × g for 10 min. The supernatant was collected as soluble protein and the pellet was collected and resuspended in the same volume as insoluble protein.

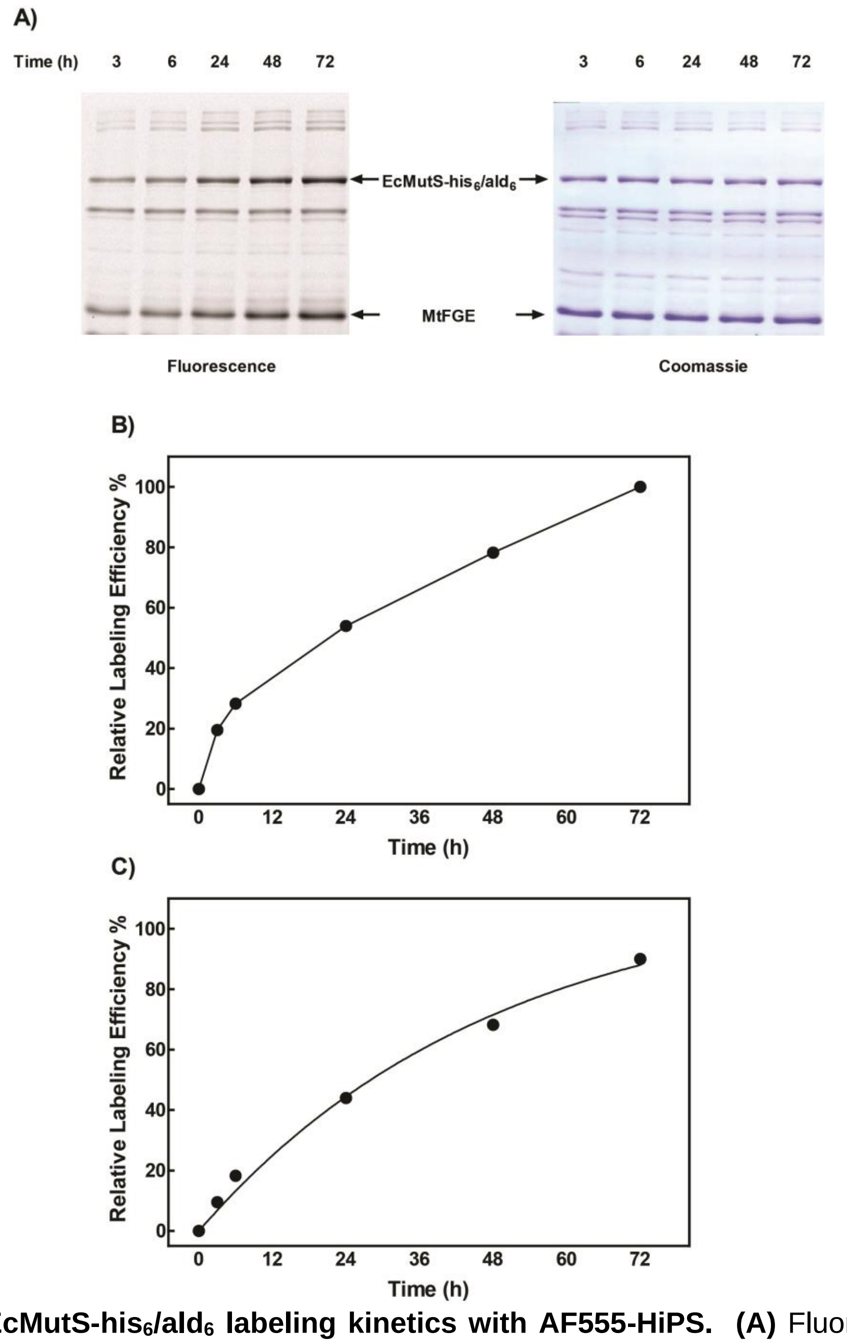

Figure S5. EcMutS-his$_6$/ald$_6$ labeling kinetics with AF555-HiPS. (A) Fluorescence and coomassie gels of EcMutS-his$_6$/ald$_6$ converted by MtFGE *in vitro* for 48h and labeled with 0.4 mM AF555 HiPS at 0°C for indicated time, and the corresponding normalized labeling efficiencies **(B)**. **(C)** Corresponding normalized labeling efficiencies subtracted by 10% for non-specific labeling.

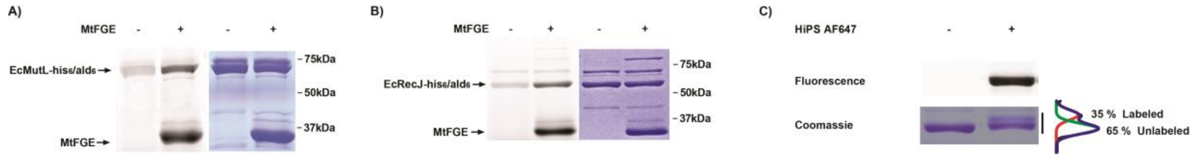

Figure S6. Fluorescent and coomassie blue stained gels of FGE conversion-dependent labeling and EcMutL(ald$_6$)-his$_6$ labeling. **(A)** FGE *in vitro* conversion-dependent HIPS-fluorophore labeling of EcMutL-his$_6$/ald$_6$. **(B)** FGE *in vitro* conversion-dependent HIPS-fluorophore labeling of EcRecJ-his$_6$/ald$_6$. Well-known auto-conversion of MtFGE results in HIPS-fluorophore labeling shown in (A) and (B). **(C)** EcMutL(346 ald$_6$)-his$_6$ labeling by 2mM AF647 HiPS for 24h after *in vitro* conversion. The use of AF647 HiPS with the EcMutL(346 ald$_6$-his$_6$) internal labeled protein allowed us to quantitatively determine labeling efficiency by separating labeled from unlabeled protein with SDS-PAGE (35% labeled; 65% unlabeled)

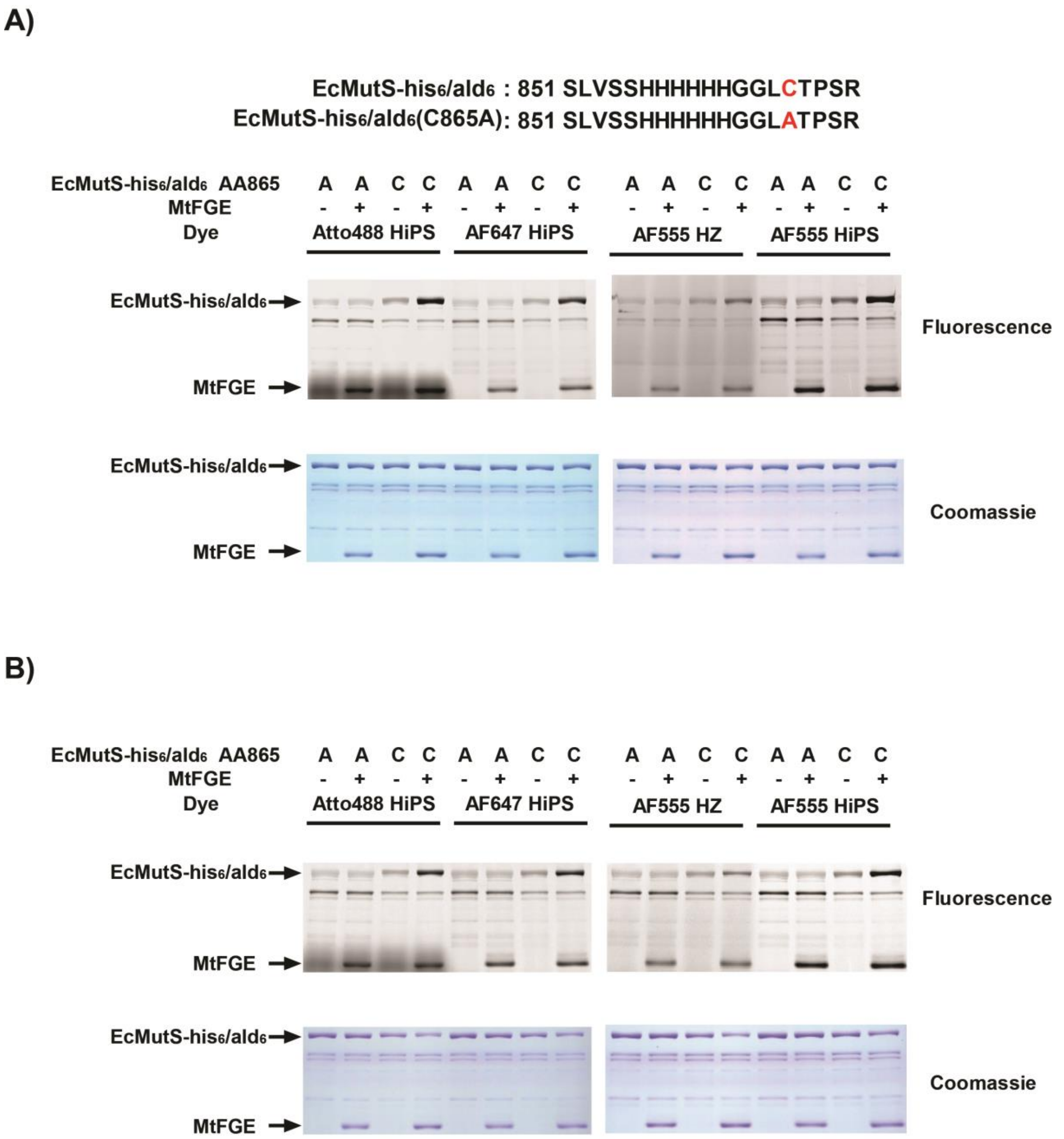

Figure S7. The specificity of HiPS fluorophore labeling with/without boiling samples. (A) Fluorescence and coomassie gels of EcMutS-his$_6$/ald$_6$ and EcMutS-his$_6$/ald$_6$(C865A) labeled by 2 mM HiPS-dyes or 3.2 mM hydrazide-dye with/without FGE conversion *in vitro*. Samples were boiled before loaded on SDS-PAGE. (B) Fluorescence and coomassie gels of EcMutS-his$_6$/ald$_6$ and EcMutS-his$_6$/ald$_6$(C865A) labeled by 2 mM HiPS-dyes or 3.2 mM hydrazide-dye with/without FGE conversion *in vitro*. Samples in B were not boiled before loaded on SDS-PAGE.

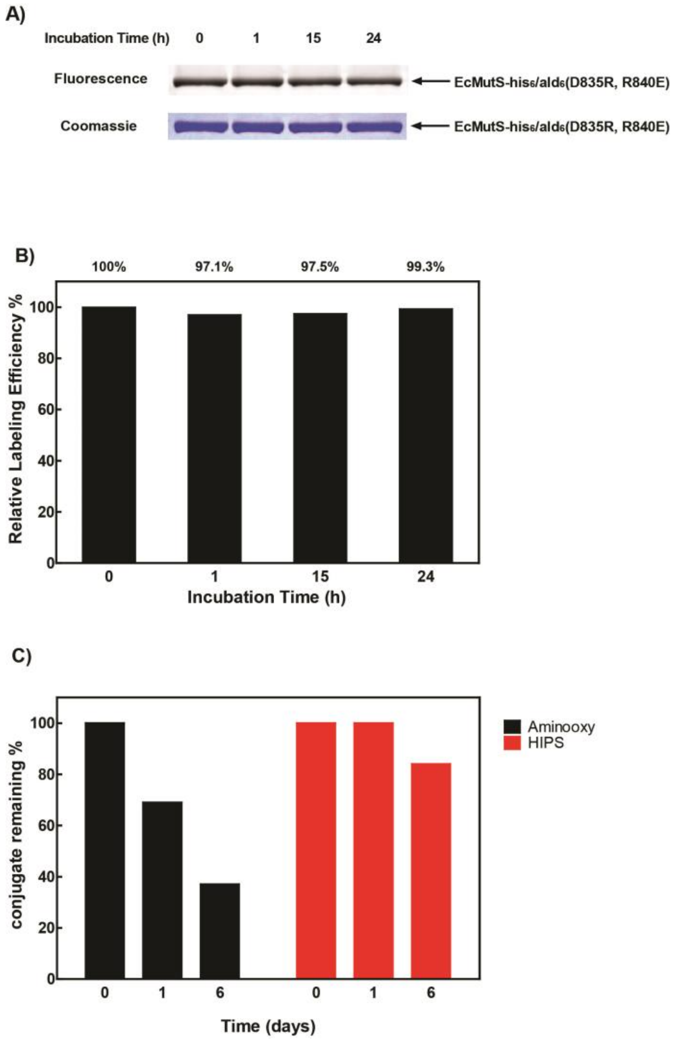

Figure S8. Hydrazino-Pictet-Spengler ligation is stable and irreversible. (A) Fluorescence and coomassie gels of purified labeled EcMutS-his$_6$/ald$_6$(D835R, R840E) after incubation for indicated time at 25 ^{o}C, and the corresponding normalized labeling efficiencies **(B)**. **(C)** Stability of αHer2-C-ald$_6$ labeled with AF594-aminooxy or AF594-HiPS. aHer2 antibody containing an aldehyde tag at the C-terminus of the heavy chain was conjugated to AF594-aminooxy or AF594-HiPS at 3.0 mg/mL with 12 AF594:antibody equivalents for 24 h at 37 ^{o}C in 150 mM NaCl and either 100 mM NaCitrate pH 4.6 (aminooxy) or 100 mM NaCitrate pH 5.5 (HiPS). Unconjugated dye was removed using Size Exclusion Chromatography (SEC; GE Healthcare Superdex 200 10/300 GL; mobile phase: PBS, pH 7.4). Isolated conjugate was incubated at 37°C for 1 or 6 days. Incubated samples were reduced with 2-Mercaptoethanol and examined on a 10% SDS-PAGE gel alongside conjugate from time 0 (no 37°C incubation). The gel was stained with Coomassie Blue to visualize total protein. Conjugation of either AF594-aminooxy or AF594-HiPS to the aldehyde-tagged antibody results in a gel shift of the antibody heavy chain. The amount of conjugate was quatitated by gel densitometry (ImageJ) and the conjugated dye determined by dividing the amount of conjugated heavy chain by the total amount of heavy chain. The amount of conjugate at each time point was calculated and compared to the amount of conjugate at time 0 to determine the relative amount of conjugate remaining at each time point.

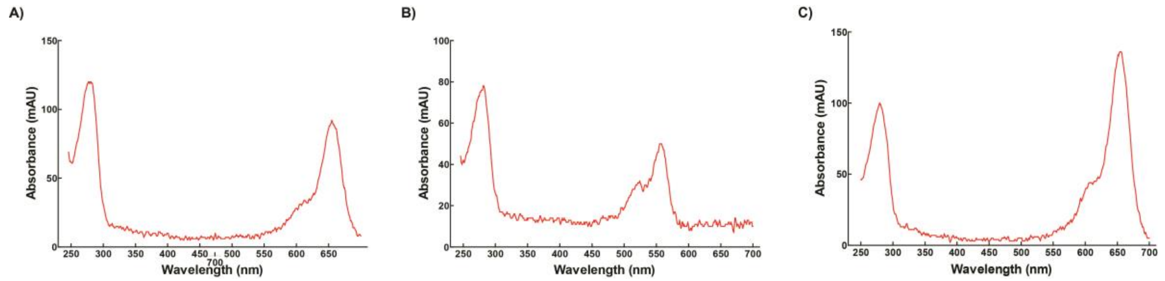

Figure S9. Spectrophotometry of purified EcMutS-his$_6$/ald$_6$ (A), EcMutS-his$_6$/ald$_6$(D835R,R840E) (B) and EcMutL-his$_6$/ald$_6$ (C) used to determine labeling efficiency.

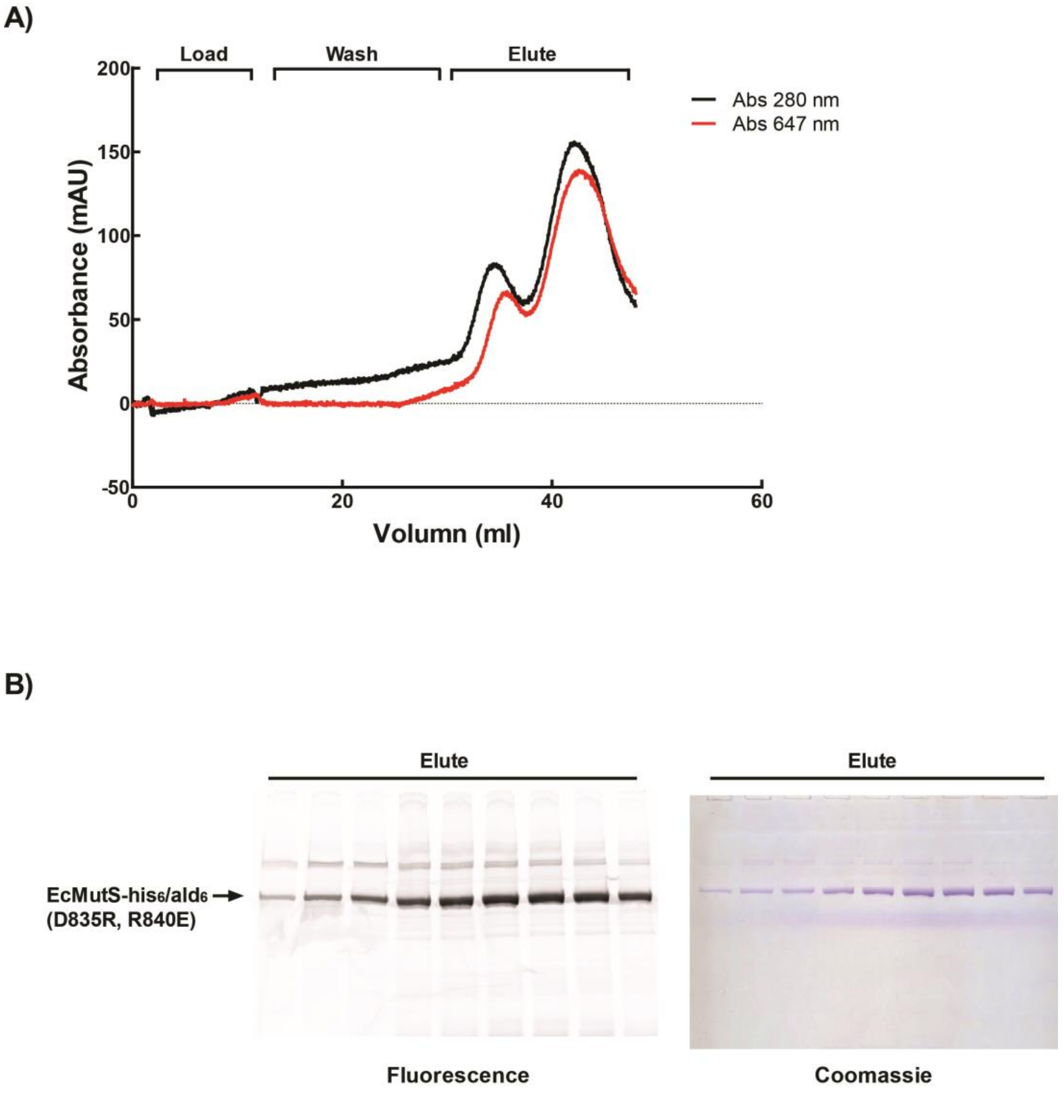

Figure S10. Hydrophobic interaction chromatography of AF647-HiPS labeled EcMutS-his$_6$/ald$_6$(D835R, R840E). **(A)** Butyl Sepharose 4 Fast Flow column purification of AF647-HiPS labeled EcMutS-his$_6$/ald$_6$ (D835R, R840E). **(B)** Fluorescence and coomassie gels of protein fractions in (A).

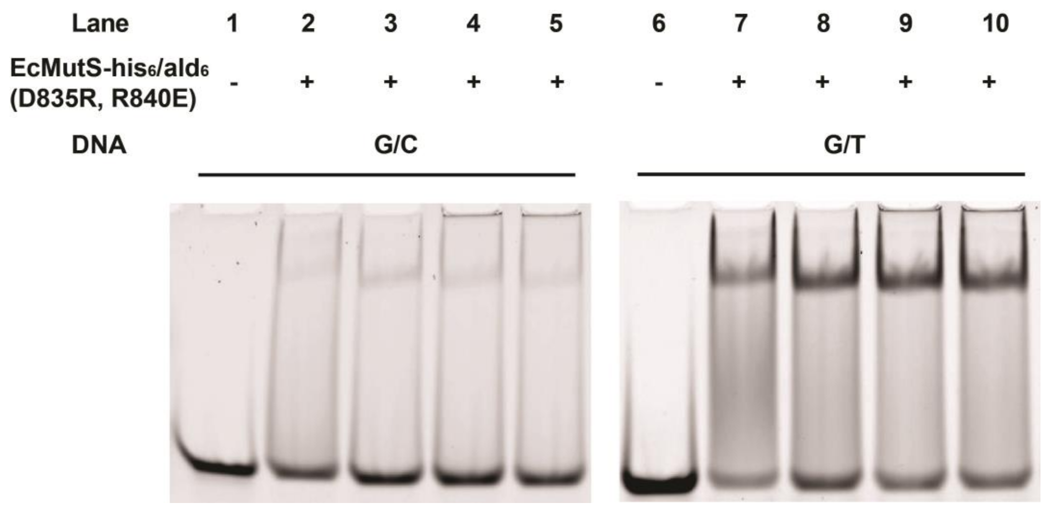

Figure S11. Gel mobility shift assays of unlabeled and fluorophore labeled EcMutS-his$_6$/ald$_6$(D835R, R840E) with FAM-labeled 41 base pair homoduplex (G/C) or G/T mismatch (G/T) oligonucleotide. EcMutS-his$_6$/ald$_6$(D835R, R840E) in lane 2, 7 were from Ni-NTA column purification and without conversion or labeling; EcMutS-his$_6$/ald$_6$(D835R, R840E) in lane 3, 8 were from Ni-NTA column purification with FGE conversion but without labeling; EcMutS-his$_6$/ald$_6$(D835R, R840E) in lane 4, 9 were converted with FGE *in vitro* and labeled with 2 mM AF555-HiPS; EcMutS-his$_6$/ald$_6$(D835R, R840E) in lane 5, 10 were converted with FGE *in vitro* and labeled with 0.5 mM AF555-HiPS.

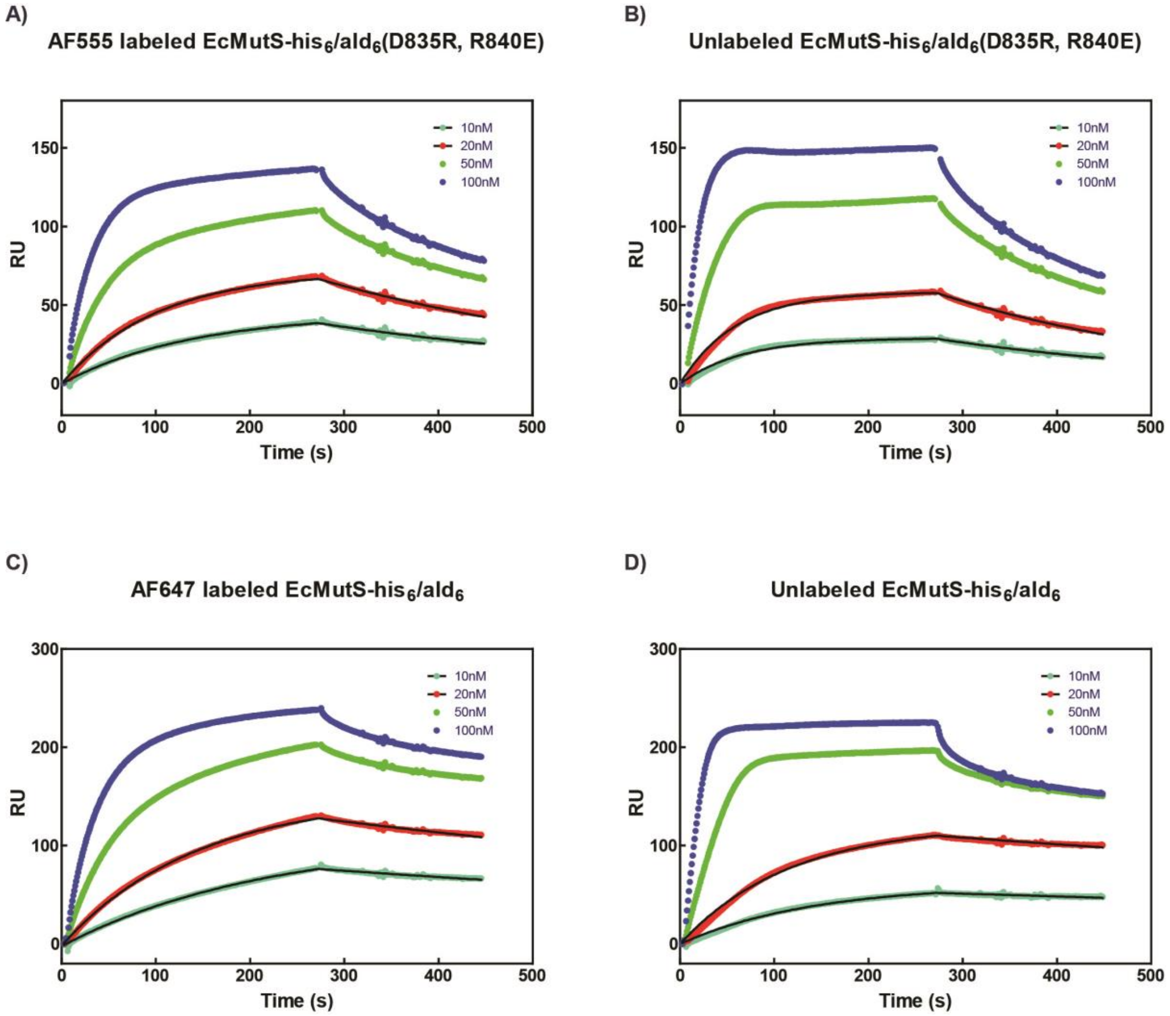

Figure S12. Mismatch DNA binding activities of EcMutS before and after fluorophore labeling. (A-D) Binding and dissociation of EcMutS with mismatched DNA is reflected by the changes in the Response Units (RU) with time. Curve fitting of 10 nM and 20 nM EcMutS concentrations are showed in black line for reference.

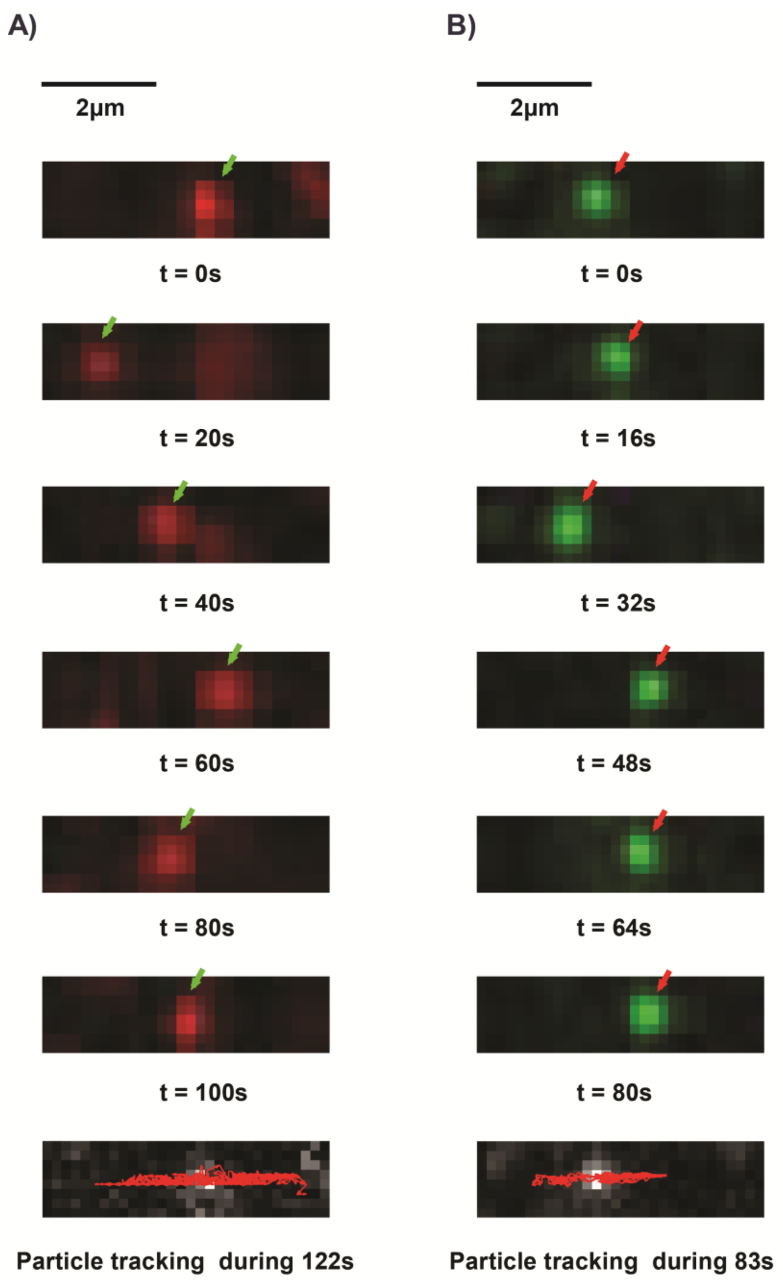

Figure S13. Single molecule tracking of fluorophore labeled EcMutS and EcMutL on a lambda DNA containing a single mismatched nucleotide. **(A)** The diffusion of HIPS-AF647 labeled EcMutS-his$_6$/ald$_6$ (red) was tracked on a single 17 Kb λ-based DNA molecule. Arrow indicates the location of EcMutS-his$_6$/ald$_6$ at indicated time points (sec). Bottom panel shows EcMutS-his$_6$/ald$_6$ particle tracking over a 120 s period. **(B)** The diffusion of HIPS-AF555 labeled EcMutS-his$_6$/ald$_6$(D835R,R840E**)** (green) was tracked on a single 17 Kb λ-based DNA molecule. Arrow indicates the location of EcMutS-his$_6$/ald$_6$(D835R,R840E**)** at indicated time points (sec). Bottom panel shows EcMutS-his$_6$/ald$_6$(D835R,R840E**)** particle tracking over a 83 s period.

Movie-1

Representative movie of ATP-bound AF647-labeled EcMutS-his$_6$/ald$_6$ sliding clamp associated with a 17 Kb λ-based DNA containing a single mismatched nucleotide. Note left-right linear movement of red-colored particle. 488 frames; 122 s.

Movie-2

Representative movie of ATP-bound AF555-labeled EcMutS-his$_6$/ald$_6$(D835R,R840E) sliding clamp associated with a 17 Kb λ-based DNA containing a single mismatched nucleotide. Note left-right linear movement of green-colored particle. 330 frames; 83 s.
